# Genetic effects on the commensal microbiota in inflammatory bowel disease patients

**DOI:** 10.1371/journal.pgen.1008018

**Published:** 2019-03-08

**Authors:** Hugues Aschard, Vincent Laville, Eric Tchetgen Tchetgen, Dan Knights, Floris Imhann, Philippe Seksik, Noah Zaitlen, Mark S. Silverberg, Jacques Cosnes, Rinse K. Weersma, Ramnik Xavier, Laurent Beaugerie, David Skurnik, Harry Sokol

**Affiliations:** 1 Centre de Bioinformatique, Biostatistique et Biologie Intégrative (C3BI), Institut Pasteur, Paris, France; 2 Department of Epidemiology, Harvard T.H. Chan School of Public Health, Boston, Massachusetts, United States of America; 3 Department of Statistics, The Wharton School at the University of Pennsylvania, Philadelphia, Pennsylvania, United States of America; 4 Department of Computer Science and Engineering, University of Minnesota, Minneapolis, Minnesota, United States of America; 5 Broad Institute of Harvard and MIT, Cambridge, Massachusetts, United States of America; 6 Center for Computational and Integrative Biology, Massachusetts General Hospital and Harvard Medical School, Boston, MA, United States of America; 7 Biotechnology Institute, University of Minnesota, St. Paul, Minnesota, United States of America; 8 Department of Gastroenterology and Hepatology, University of Groningen and University Medical Center Groningen, Groningen, the Netherlands; 9 Department of Gastroenterology, Saint Antoine Hospital, Paris, France; 10 Department of Medicine, University of California, San Francisco, California, United States of America; 11 Zane Cohen Centre for Digestive Diseases, Mount Sinai Hospital, Toronto, Ontario, Canada; 12 Sorbonne Université, Paris, France; 13 Division of Gastroenterology, Massachusetts General Hospital and Harvard Medical School, Boston, Massachusetts, United States of America; 14 Division of Infectious Diseases, Harvard Medical School, Boston, Massachusetts, United States of America; 15 Massachusetts Technology and Analytics, Brookline, Massachusetts, United States of America; 16 Department of Microbiology, Necker Hospital and University Paris Descartes, Paris, France; 17 INSERM U1151-Equipe 11, Institut Necker-Enfants Malades, Paris, France; 18 Micalis Institute, AgroParisTech, Jouy-en-Josas, France; 19 INSERM CRSA UMRS U938, Paris, France; Stanford University, UNITED STATES

## Abstract

Several bacteria in the gut microbiota have been shown to be associated with inflammatory bowel disease (IBD), and dozens of IBD genetic variants have been identified in genome-wide association studies. However, the role of the microbiota in the etiology of IBD in terms of host genetic susceptibility remains unclear. Here, we studied the association between four major genetic variants associated with an increased risk of IBD and bacterial taxa in up to 633 IBD cases. We performed systematic screening for associations, identifying and replicating associations between *NOD2* variants and two taxa: the *Roseburia* genus and the *Faecalibacterium prausnitzii* species. By exploring the overall association patterns between genes and bacteria, we found that IBD risk alleles were significantly enriched for associations concordant with bacteria-IBD associations. To understand the significance of this pattern in terms of the study design and known effects from the literature, we used counterfactual principles to assess the fitness of a few parsimonious gene-bacteria-IBD causal models. Our analyses showed evidence that the disease risk of these genetic variants were likely to be partially mediated by the microbiome. We confirmed these results in extensive simulation studies and sensitivity analyses using the association between NOD2 and *F*. *prausnitzii* as a case study.

## Author summary

In this study, we used observational data to explore associations between host genetics and the commensal microbiome in inflammatory bowel disease cases. Our analysis identified four associations involving two genes and four bacterial taxa and replicated two of these associations in independent cohorts. Then, we developed a counterfactual framework to assess the fitness of a few parsimonious gene-bacteria-IBD causal models. These analyses confirmed the robustness of the identified associations and highlighted microbiota mediation as a potential mechanism underlying the association between IBD and those genetic variants while ruling out reverse causation and arguing against a bacteria-IBD association resulting from a shared genetic effect.

## Introduction

Most genetic analyses and twin studies published to date support a genetic component of inflammatory bowel disease (IBD) phenotypes [1]. Recent works showed an estimated heritability on the liability scale of approximately 70–80% for Crohn’s disease (CD) and 60–70% for ulcerative colitis (UC) [2]. Genome-wide association studies (GWASs) have identified more than 200 loci associated with IBD, most of which are shared between UC and CD [3–5]. Identification of these loci has enhanced our understanding of the pathogenesis of IBD, provided perspective on key pathways, and highlighted an essential role for host defense against infection. In parallel, recent work noted a potential role of the intestinal microbiota in initiating, maintaining, and determining IBD-related phenotypes [6–8]. Although generally the commensal microbiota is accepted to induce inappropriate activation of intestinal mucosal immunity, the precise role of the microbiota in the etiology of IBD in terms of host genetic susceptibility remains unclear.

Both functional and observational studies have been performed in an expanding effort to address the question of interplay between human genetic variation and the gut microbiome. In a recent work in mice, we showed that the IBD-associated gene *CARD9* affected the composition and function of the gut microbiota by altering the production of microbial metabolites and increasing the risk of intestinal inflammation [9]. Similarly, another study explored potential gene-microbiota interactions in the pathogenesis of IBD [10]. The study showed that the IBD-associated genes *ATG16L1* and *NOD2* most likely played an essential role in the beneficial immunomodulatory properties of *Bacteroides fragilis*, which protects mice from experimental colitis. A number of systematic screenings with observational data from healthy human subjects have also been performed using various strategies. In particular, three groups performed population-based GWASs of 1,561, 1,514 and 1,812 individuals [11–13]. Overall, these studies confirmed an association between host genetics and the human gut microbiome composition. Comparison of results between studies also highlighted a probable complex genetic architecture underlying microbiome traits, with little overlap among the detected loci between studies [14]. Alternatively, at least one GWAS performed with 474 IBD cases identified an association between *NOD2* variants and the relative abundance of *Enterobacteriaceae* [15].

Altogether, these studies have improved our understanding of the relationship between host genetics and the human microbiome. However, functional studies and most *in vitro* experiments may fail to replicate the precise conditions of the organism under study [16]. Moreover, many species are difficult to grow *in vitro*, although progress has been made recently [17, 18]. On the other hand, existing observational studies have focused only on association testing and have neither leveraged the multidimensional aspect of the data nor accounted for the study design to assess causal relationships. Here, we aimed to assess whether single nucleotide polymorphisms (SNPs) from four major genes (*NOD2*, *CARD9*, *LRRK2*, and *ATG16L1*) associated with IBD were also associated with bacterial populations from the gut and to evaluate the relevance of the underlying causal models. We used a discovery dataset including 182 IBD cases with both microbiome and host genetic data as well as replication data from 451 additional IBD cases. First, we applied *CMS*, a novel association mapping approach we recently developed to screen for gene-bacteria associations [19]. Then, we developed an inference framework based on the counterfactual principle [20] to assess the fitness of a few parsimonious gene-bacteria-IBD models with the observed associations.

## Results

### SNP-bacteria association screening

**Fig 1:**
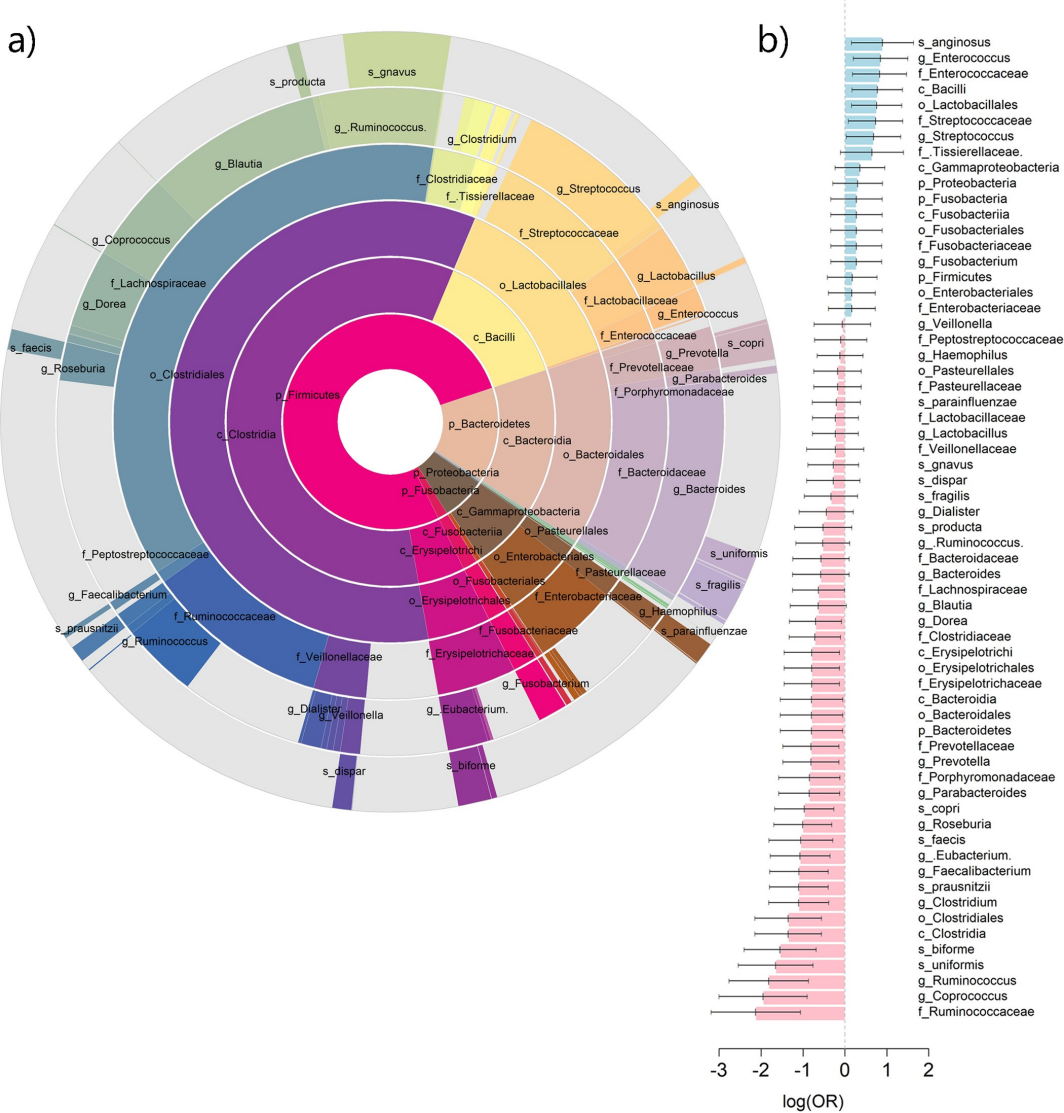
Overview of the bacterial taxa distribution in IBD cases and NOD2 association. Panel a) shows the relative proportion of the 168 bacterial taxa analyzed across the six hierarchical levels. Only taxa representing more than 0.5% of the total bacterial loading are labelled. Grey areas correspond to unknown, unmeasured, or underrepresented taxa. Panel b) show the log of the odds ratio (OR) of the labelled bacteria on IBD case-control status and corresponding 95% confidence interval, as derived using standard logistic regression while adjusting for confounding factors.

Our discovery dataset included variants from four genes (*NOD2*, *CARD9*, *LRRK2*, and *ATG16L1*, **S1 Table**) and bacterial levels from the gut obtained through 16S sequencing of 182 well-phenotyped IBD cases (**Table 1**). After stringent quality control (**S1 Text**, **S1 Fig**), 168 bacterial taxa remained for association testing. The relative proportion of these 168 taxa within each level is summarized in **Fig 1A**. A total of 6, 12, 17, 36, 63, and 34 taxa were included in each of the 6 hierarchical levels (phylum, class, order, family, genus and species, respectively). The bacterial microbiota was dominated by bacteria from the *Firmicutes*, *Bacteroidetes* and *Proteobacteria* phyla. When compared to a similar cohort of 38 healthy controls (who had complete microbiome data but no SNP data) using standard logistic regression (**S2 Fig**), we found that 73% of those bacteria displayed a negative association with IBD (**Fig 1B**).

**Table 1:** Sample characteristics.

	Female		Male		Total
	CD	UC	CD	UC	
**N**	67	38	48	29	182
**Flare (%)**	40.3%	55.3%	50.0%	41.4%	46.2%
**Mean age**	40.3	38.4	39.9	45.5	40.6
**Smoking (%)**	25.4%	7.9%	31.3%	13.8%	21.4%
***Treatment***					
**Oral 5-ASA (%)**	20.9%	55.3%	35.4%	62.1%	38.5%
**Corticosteroids (%)**	11.9%	23.7%	16.7%	31.0%	18.7%
**Anti-TNF (%)**	56.7%	44.7%	54.2%	31.0%	49.5%
**Thiopurine-MTX (%)**	43.3%	31.6%	29.2%	51.7%	38.5%

Abbreviation: MTX, methotrexate.

We performed systematic screening for associations between the four IBD-susceptibility genetic variants and bacterial quantification. Due to substantial correlation across hierarchical levels (**S3 Fig**), we conducted analyses within each level and applied Bonferroni correction to the *p*-value within each level to select the best candidate associations. An association test was performed using standard linear regression after adjusting for established confounding factors as well as covariates selected by the recently developed *CMS* approach [19] to increase the statistical power (**S1 Text**). We identified seven candidate associations (**S2 Table**), six of which involved *NOD2* (with *c_Bacteroidia*, *f_Bacteroidaceae*, *g_Bacteroides*, *g_Roseburia*, *R*. *faecis*, and *F*. *prausnitzii*) and one that involved *CARD9* (with *p_Firmicutes*). However, these associations corresponded to four independent signals (**Table 1**) with the same association being projected along branches of the phylogenic tree (see **S4–S7 Figs** for hierarchical plots of the associations). Indeed, *g_Roseburia* and *R*. *faecis*, which were found to be associated with *NOD2*, had a correlation of 0.84 in our dataset. Similarly, *f_Bacteroidaceae* and *g_Bacteroides*, which were also associated with *NOD2*, were almost identical and had a correlation of 0.93 with *c_Bacteroidia*. The two remaining associations were observed between *NOD2* and *F*. *prausnitzii* and between *CARD9* and *p_Firmicutes*. All four signals showed that the IBD risk alleles (of either *NOD2* or *CARD9*) were negatively associated with the bacteria in question.

**Fig 2:**
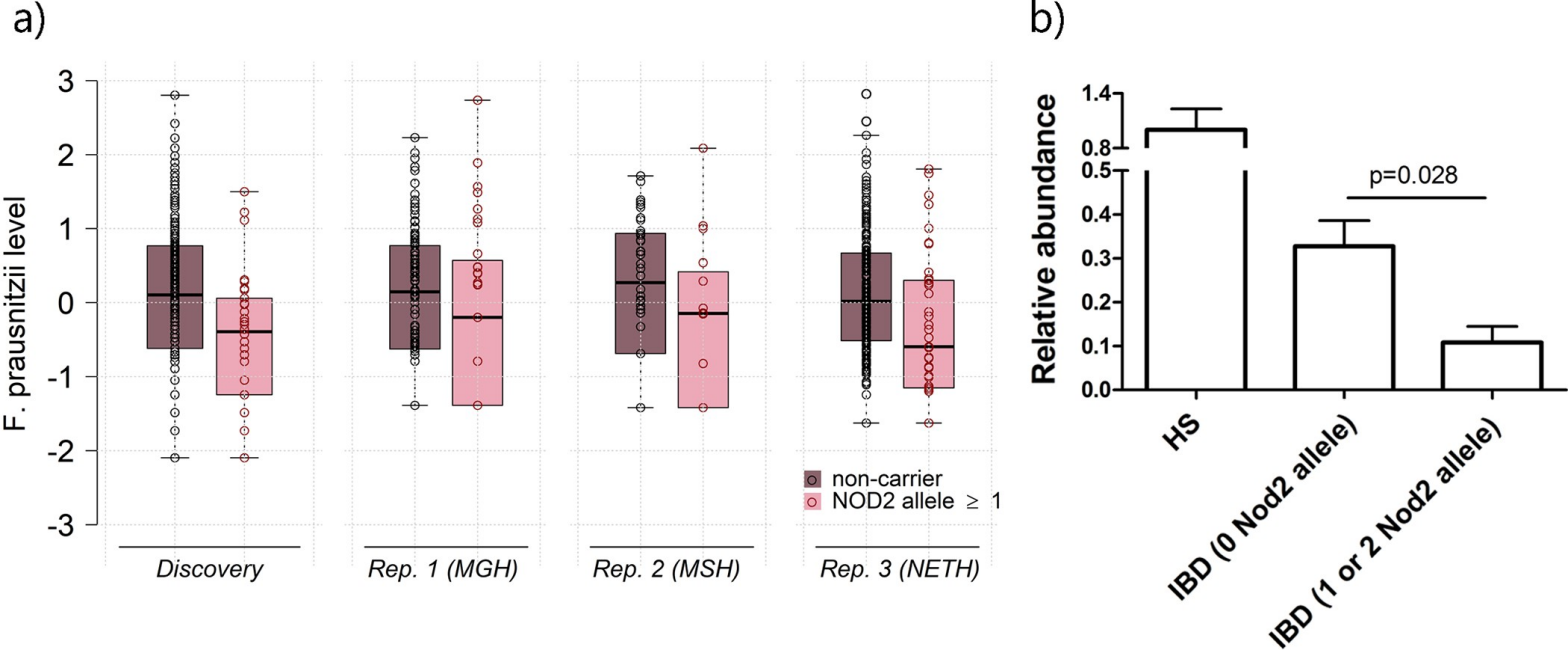
Summary of association signals between NOD2 variants and F. prausnitzii. Panel a) presents the boxplots of rank-based inverse normalized *F*. *prausnitzii* from fecal microbiota in the discovery samples and the three replication datasets for non-carrier and carriers of NOD2 risk alleles. Panel b) presents *F*. *prausnitzii* in the healthy controls and cases from the discovery sample quantified using 16S qPCR and normalized to the global bacterial population (mean ± s.e.m.).

To validate these associations, we performed in silico replication using three independent datasets of adult human subjects with IBD including a total of 451 individuals [15, 21]. Genetic variant and bacteria data were only partly available in these datasets. *CARD9* was available in only one of the three replication cohorts, and we used the genus *g_Faecalibacterium* as a proxy for *F*. *prausnitzii* in all three replication analyses. We preprocessed and analyzed the data similarly to that of the discovery sample and combined the two stages using a standard inverse-variance meta-analysis (**Table 1**). The replication analysis confirmed the associations between the *NOD2* risk allele and decreased abundance of *g_Roseburia* and *F*. *prausnitzii* (**Fig 2A**); and both signals passed a stringent Bonferroni correction accounting for all tests performed at the meta-analysis stage (*P* < 7x10^-5^). *Firmicutes-CARD9* and *Bacteroides-NOD2* replication showed a signal concordant with those observed during discovery (i.e., a negative association) but were not significant, indicating either a false positive in the discovery or a lack of statistical power in the replication. To explore further potential heterogeneity of the SNP effects, we conducted association analyses for the following disease subtypes: CD overall, Ileal CD, non-ileal CD, and ulcerative colitis (**Table 1**). The effect estimates for CD and ileal CD were consistent with the signal observed for IBD during both discovery and replication. Conversely, we observed heterogeneity of effects for both non-ileal CD and UC. Finally, we confirm further the *NOD2* and *F*. *prausnitzii* association, which is a bacterium of particular interest for our group[22], using real-time quantitative PCR [23] in the discovery samples (**Fig 2B**).

### Model fit while accounting for selection bias

**Fig 3:**
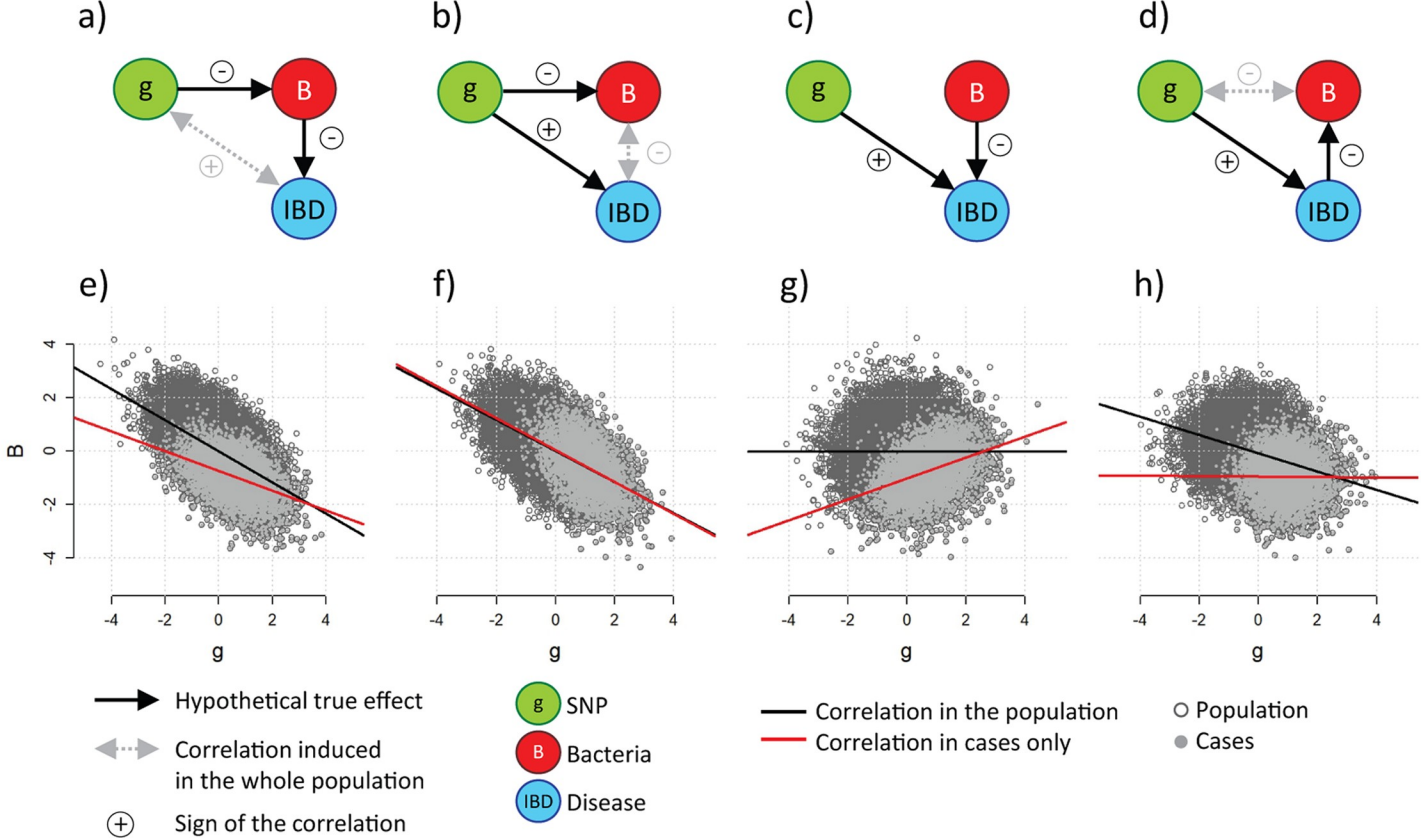
Hypothetical causal models. Expected correlation between an IBD risk variant *g* and a bacteria *B* negatively correlated with IBD, across four potential underlying models. For illustrative purposes, *g* and *B* are assumed to be continuous and normally distributed and all effects are larger than those observed in real data. Top panels (a, b, c, and d) present the hypothetical causal diagrams and bottom panels (e, f, g, and h) present the corresponding scatterplots of *B* as a function of *g* in the population (dark grey points, and trend in black), and in cases only (light grey points and trend in red). In model a) the effect of *g* on IBD is mediated by *B*; in cases the effect of *g* on *B* is underestimated because of the oversampling of participants carrying risk alleles (e). In model b) the genetic variant influences both IBD and *B*, inducing a correlation between IBD and *B* which is observed in both the whole sample and cases only (f). In model c), *g* and *B* act independently on IBD and are therefore not associated in the population, however *g* and *B* are positively correlated in case-only samples because of biased selection (g). Finally, in model d) the effect of *g* on *B* is mediated by IBD; the indirect association between *g* and *B* observed in the general population is canceled when looking at cases only as the sample is stratified by the mediator (h).

The associations between the IBD risk variants and two bacteria that are also associated with the IBD risk raise questions about potential reverse causation or conversely potential mediation of the genetic risk through bacteria. In an attempt to address the question of causality, we performed a series of analyses to assess the fitness of models matching existing knowledge, which can be summarized as follows. First, the effects of both *NOD2* and *CARD9* on IBD are well established [3]. Second, SNPs can only be explanatory variables, which implies a (direct or indirect) unidirectional relationship. Third, the four taxa from **Table 1** (*Roseburia* genus, *Bacteroidia* class, *F*. *prausnitzii*, and *Firmicutes* phylum) have been consistently found to be negatively correlated with the IBD case status [8, 24]; however, the directionality of the effect remains a topic of investigation [25]. In regards of these three constraints, four (potentially overlapping) parsimonious underlying causal models can fit the data (**Fig 3A–3D**): (a) the effect of the risk allele is mediated by bacterial level; (b) the genetic variant influences both the disease and the bacteria, inducing a correlation between the two latter variables; (c) the risk allele and bacteria are independent risk factors of IBD; and (d) IBD mediates the effect of the risk allele on bacterial level (a so-called reverse causation). Note that these models remain relevant when adding intermediate factors. For example, *NOD2* variants have been shown to correlate with lower concentrations of *defensins* [26], which in turn can be responsible for changes in the microbiota composition. Alternatively, IBD may be associated with increased epithelial oxygenation [27], which itself can be responsible for changes in the microbiota composition. Such scenarios will be in full agreement with model a) and model d), respectively, and therefore will not alter the conclusion of the analysis.

Importantly, secondary phenotype analysis in highly ascertained samples (such as in the present study which includes IBD cases only) can introduce selection bias [28, 29], although previous work has shown that this bias will be minimal for rare diseases (*i*.*e*., those with a prevalence ≤ 1%, such as IBD [30]) and for cases with low to moderate associations between variables [29] (**S3 Table**). To assess the fitness of the data with the four causal models while accounting for selection bias, we derived an estimator of Δ, the bias induced by the IBD case-only sampling in the regression coefficient between the risk allele tested and the bacteria. As shown in **S1 Text**, Δ can be approximated in some specific situations (common diseases and modest effects of the predictors), but estimation in more general cases is extremely challenging. Nevertheless, this theoretical framework allowed us to determine the expected sign of the risk allele-bacteria association across the scenarios considered without relying on assumption on the data distributions. Following the counterfactual principle [20], we leveraged this property to infer which model had the highest fit for the data based on the signs of the observed associations. As illustrated in the toy example from **Fig 3E–3H**, our analytical derivation (see Materials and Methods) showed that the ascertainment could induce a positive bias of the SNP-bacteria association in model (c) and lead to the removal of the SNP-bacteria association in model (d). The results from this analysis are twofold. First, it shows strong evidence that among the four causal models considered, models (a) and (b), which included a true gene-bacteria effect, were the only models in which we expected a persistent negative SNP-bacteria effect, which was in agreement with the results from **Table 1**. Second, it rules out selection bias as a likely explanation for the observed association.

**Fig 4:**
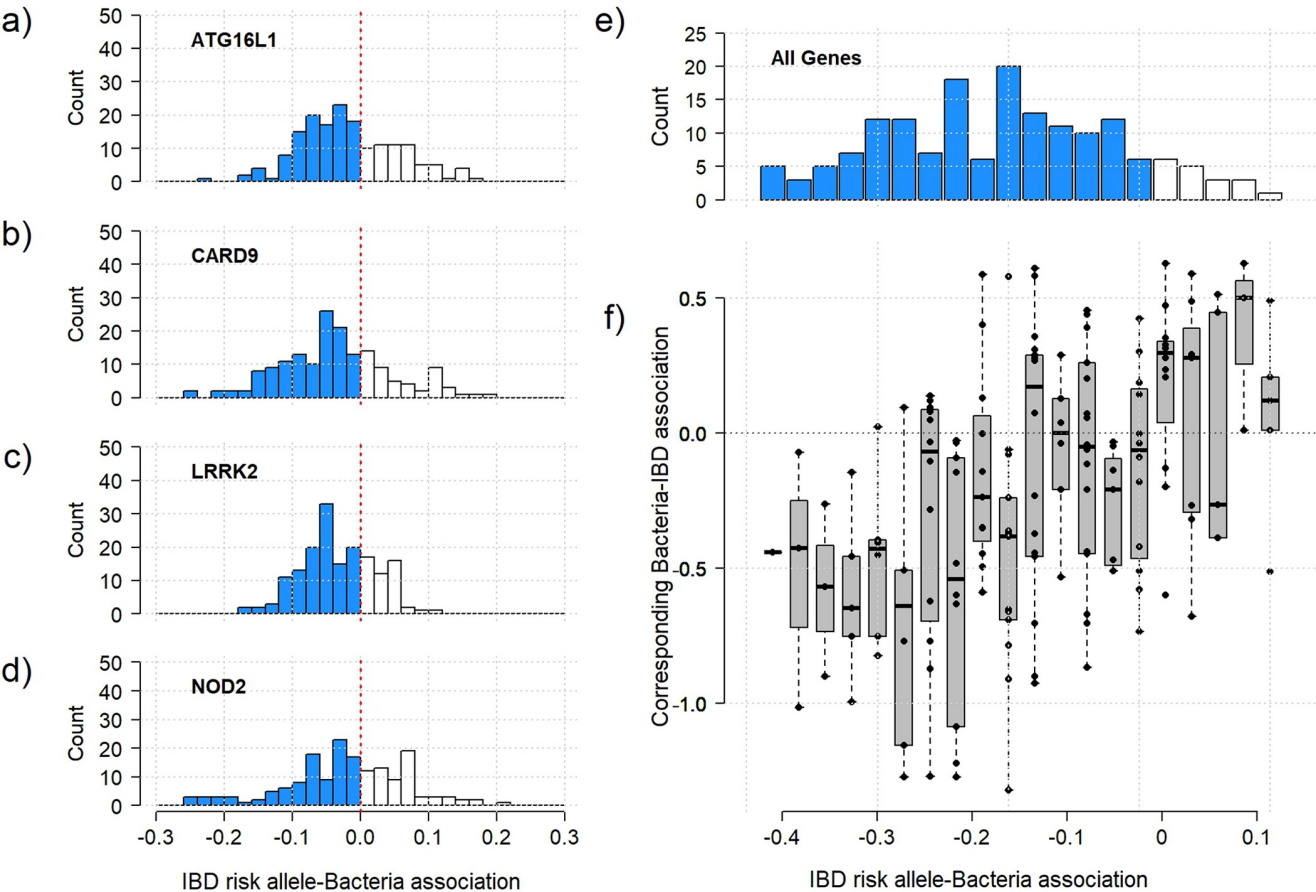
Correlation between SNP-taxa and IBD-taxa effect estimates. The 168 bacterial taxa were tested for association with the variants from each of the four genes considered: (a) ATG16L1 (rs12994997), (b) CARD9 (rs10781499), (c) LRRK2 (rs11564258), and (d) NOD2 (rs2066844, rs2066845, and rs2066847). The histograms on the left panel show the distribution of IBD risk alleles-bacteria association (i.e. of ${\hat{\beta }}_{g}$, the regression coefficients) and the enrichment for negative effects (in blue, p-values equal 7.1x10^-5^, 3.3x10^-8^, 1.3x10^-8^, and 5.3x10^-3^, respectively). Panel (e) shows a similar histogram while merging the per-risk allele change in bacteria level of the four genes (i.e. summing for each bacteria the ${\hat{\beta }}_{g}$ of the four genes). Panel (f) shows the distribution of bacteria-IBD association derived in a IBD cases-controls dataset (${\hat{\beta }}_{B}$) for each bin from panel (e). Together, panels (e) and (f) show the strong concordance of the gene-bacteria and bacteria-IBD effects, in agreement with a mediation effect of the risk allele on IBD through the microbiome. In particular, bacteria displaying lower level in carrier of IBD risk alleles are more likely to be negatively associated with the risk of IBD.

We then asked whether the proposed approximation of Δ could be also informative for the remaining gene-bacteria association results. Indeed, besides the main signals from **Table 1**, our data showed both enrichment for a negative bacterium-disease association (**Fig 1B**) and significant enrichment for negative effects of IBD risk alleles on all bacteria levels, with 65%, 71%, 70%, and 60% of the associations negative for *ATG16L1*, *CARD9*, *LRRK2*, and *NOD2*, respectively (**Fig 4A–4D**, *p*-values for enrichment equal 7.1x10^-5^, 3.3x10^-8^, 1.3x10^-8^, and 5.3x10^-3^, respectively). After aligning the bacterium-disease and bacterium-SNP associations, we observed a strong and significant (*P* = 5.0x10^-8^) concordance of effects (i.e., most bacteria showed an association with SNPs and IBD with the same sign) (**Fig 4E and 4F**). However, our derivation and simulation study (**S1 Text** and **S8–S12 Figs**) demonstrated that, regardless of the error distribution of the bacterial level, the correlation between the bacterium-disease and the bacterium-SNP effect estimates in the case-only samples, is expected to be negative under model c), and null under model d). Therefore, the observed positive correlation (**Fig 4F**) provides further evidence against model c) (the risk allele and bacteria act independently on IBD) and d) (IBD mediates the effect of the risk allele on the bacteria) as generative models, and again indicates that models a) and b) are the best fits for our data.

Fully deciphering models a) and b) would be extremely challenging because of the complex interrelationships between the variables in question and the untestable assumptions that would have to be assumed. However, we found some indications supporting model a) against model b) in our data. In particular, as illustrated in **Fig 3**, the association effect estimates between a genetic variant and bacteria are expected to be slightly deflated in the cases alone compared to that of the estimates derived in a population including controls in model a) but should remain unchanged in model b). To assess the fitness of the two models, we use the special case of *NOD2*, which is associated almost exclusively with CD [3]; thus, the *NOD2*-bacteria associations are expected to be unchanged between all IBD samples (including UC cases) and CD alone under model b) but should be different in model a). As shown in **S13 Fig** for all bacteria, we found that the effect estimates derived for the CD cases alone were significantly (*P* = 5x10^-3^) smaller than those derived for the complete dataset, which matched the expected deflation observed in the presence of mediation and therefore provided an argument in favor of model a).

### Sensitivity analyses

As most causal inference approaches, the proposed counterfactual strategy we used in this study has some limitations. In order to assess the validity of our simulation framework, we performed a series of sensitivity analyses that closely mimicked current knowledge on IBD associations. First, empirical bacterial levels follow a non-negative, overdispersed distribution and often harbor a large number of zero values; therefore, use of the Poisson distribution, negative binomial distributions, or hurdle models has been suggested for analyses of microbiome data [31]. Moreover, we applied a rank-based inverse-normal transformation in our real data analysis, although sparsity remained (after QC, the bacteria had a minimum of 20% nonzero values). To assess the impacts of these parameters, we performed a simulation study in which the bacterial levels were drawn from a negative binomial distribution with varying percentages of zero-values [0%; 95%]. For each replicate, the SNP-bacteria effect was estimated similar to the procedure used in the real data analysis. As shown in **S14 Fig**, no qualitative difference was found between the untransformed and transformed analyses over the four scenarios considered (i.e., **Fig 3A–3D**). Overall, both the true effect (models (a) and (b)) and bias (model (c)) decreased toward the null with the increased data sparsity.

Another potential limitation of our inference was that we assumed that all effects were homogeneous, and Crohn’s disease and ulcerative colitis cases were treated as a single outcome variable (IBD). However, as aforementioned, *NOD2* variants and *F*. *prausnitzii* are mostly associated with CD, and ileal CD in particular, but show little evidence of an association with UC [3]. Such effect heterogeneity can be treated as a misclassification problem (*i*.*e*., UC cases are misclassified as CD cases). To assess the impact of genetic heterogeneity, we performed a second simulation in which the SNP-disease and bacteria-disease associations were present only for a subset of the cases (thus mimicking UC-CD heterogeneity). In this simulation, all parameters were set to ensure that the magnitudes of the known effects were similar to those reported in the literature for the *NOD2*-*F*. *prausnitzii* example and in our analysis. As described above, we considered the four causal models from **Fig 3** and estimated for each replicate and each disease subtype strata the association between the genotype and the bacteria. As shown in **S15 Fig**, in this scenario, some of the bias from models (c) and (d) illustrated in **Fig 3G and 3H** decreased toward the null. Moreover, our simulations showed that under models (c) and (d), we expected no association signal from either the disease subtype strata or the whole IBD-cases sample. Again, only models a) and b) fit the observed results from both **Table 1** and **Fig 4**.

Finally, the validity of some of our analyses relies on the assumption that no intermediate variable buffers the associations with IBD. For example, genetic variants and bacteria may be associated with IBD through the disease severity instead of the disease itself. In **S16 Fig**, we repeat the simulation from **Fig 3** after replacing IBD with the disease severity drawn from a binomial distribution with *n* = 5. When assessing the relationships among IBD (here defined as severity ≥ 1), bacteria and genetic variants, we observed the same qualitative results for models a), b), and c) as those from our primary example. However, in model d), substantial correlation between genetics and bacteria persisted in the case sample. Nevertheless, if the severity is measured, a naïve but efficient solution consists of adjusting for that variable. To the best of our knowledge, no standard severity score is available for IBD. However, severity is commonly quantified by ineffective treatment, remission, or the number and location of inflamed sites. However, only the flare/remission status was available in our study. To assess potential confounding, we also reran the SNP-bacteria and bacteria-IBD associations. As shown in **S17 Fig**, we did not observe any qualitative change in the association pattern.

## Discussion

In this study, we explored the effect of four major genes associated with an increased risk of IBD on bacterial populations from the gut. First, we identified and replicated in independent cohorts the associations between *NOD2* risk alleles and a decrease of both *F*. *prausnitzii* and *g_Roseburia*. Using a counterfactual framework, we provide further evidence of the robustness of these associations and highlight potential mediation of the increased risk of IBD due to those genes by the gut microbiome. Importantly, our framework only compares a set of parsimonious models and thus provides only probable causes for the observed effects. Moreover, potential mediation effects do not rule out the presence of other mechanisms through other pathways acting in parallel. Nevertheless, the extensive sensitivity analyses, which simulated data in which the bacterial data distributions were overdispersed and sparse, and genetic effect was heterogeneous, confirmed our results. Our inference is also in agreement with previous functional studies showing that numerous IBD susceptibility genes are involved in the response to microorganisms [3], suggesting that the functional consequences of their alterations may be directed toward the gut microbiota. Indeed, studies in mice showed that defects in innate immunity genes, such as *NOD2* or *CARD9*, impacted the microbiota composition [9, 32, 33]. Moreover, the gut microbiota of *NOD2-/-* and *CARD9-/-* mice has pro-inflammatory effects by itself, because it worsens the colitis severity when transferred to germ-free wild type mice [9, 34]. Previous work also noted an essential role for *NOD2* in the temporal development and composition of the host microbiota in mice [35]. In human patients with IBD, a significant association between the *NOD2* risk allele and the fecal abundance of Enterobacteriaceae has been shown[15]. Finally, in another study, homozygocy for the *ATG16L1* risk allele was associated with increased numbers of *Fusobacteriaceae* in the inflamed ileal mucosa of CD patients [36]. Building on these previous reports, our study addresses an important missing piece by describing the role of likely interactions between human genetic variants and the gut microbiota in IBD pathogenesis.

Although our study provides evidence against some generative models, including reverse causation by which the IBD status itself influences the microbiome composition, we fully acknowledge that strong statements about causality are impossible. Instead, we argue that our analysis may at least provide support for mediation of genetic effects on IBD through the microbiome for these variants. Whether other IBD variants display similar patterns remains to be assessed. Moreover, despite extensive sensitivity analyses, other complex mechanisms may impact the validity of our results. For example, we cannot fully rule out potential confounding by disease severity. A strong interaction effect between the genetic variants considered and other IBD risk factors (i.e., where the magnitude or even the direction of the genetic effect depends on other factors) may also impact our results. The Mendelian randomization (MR) principle, which is now commonly applied to genome-wide summary statistics data [37–39], may provide additional support for potential causal models. Importantly, the proposed parsimonious models most likely match the first two assumptions of MR [i.e., i) the genetic variant is independent of typical confounding factors and ii) the genetic variant is associated with the mediator in question (here the bacterial level)]. However, the third assumption (i.e., absence of an effect of the genetic variant on the disease conditional on the mediator) potentially is violated for IBD, which will make MR analyses more challenging. Therefore, the fundamental question about correlation or causation [40] will require a range of additional analyses to be fully addressed.

Using IBD-case samples to explore gene-bacteria associations has some advantages over other study designs. Studies of healthy subjects can only be used to test for associations between genetic variants and bacteria but cannot be used to decipher potential causal models of diseases. On the other hand, although prospective cohorts in the general population have the potential to circumvent a number of the statistical artifacts we addressed in our study, they require an unrealistic sample size when studying relatively rare diseases, such as IBD. Importantly, case-control data will not solve the selection bias issue. Instead, we suggest that future studies may optimize other components of the study design, especially reducing heterogeneity of treatment and heterogeneity of disease status across individuals, and measuring additional potential risk factors of IBD. Association studies of IBD-associated variants with the microbiome of healthy participants may also be of interest. At least one previous study explored this question but did not report any specific enrichment for association of IBD variants [12]. Nevertheless, by merging summary statistics from the available *NOD2* variants (rs2066845 and rs2066845) from this study, we observed signals going in the same direction (*g_Roseburia z = -1*.*11*; *F*. *prausnitzii z = -0*.*79*), although neither signal was nominally significant (*P = 0*.*27* and *P = 0*.*43*, *respectively*). Finally, we merged all three *NOD2* variants into a binary variable defined as the presence/absence of at least one variant. Although the effect of each of these variants is established, their combined effect is not well defined [41–43]. Understanding the relative contribution of each *NOD2* variant to the overall signal we observed is of primary interest but will again require a larger sample size.

Here, we showed that part of the effect of IBD-risk variants was most likely mediated by an effect on the bacterial microbiota. The identification of this potential causal pathway from genetic variants to IBD in observational human data is important for understanding the pathogenesis of IBD and other microbiota-driven diseases. Moreover, the findings suggest that targeting the microbiota may be an effective therapeutic strategy to overcome gene-induced disease susceptibility.

## Materials and methods

### Patients and samples collection

All subjects were recruited in the Gastroenterology Department of the Saint Antoine Hospital (Paris, France) and provided informed consent, and approval was obtained from the local ethics committee (Comite de Protection des Personnes Ile-de-France IV, IRB 00003835, Suivitheque study). Their microbiota composition analysis has been published[44]. A diagnosis of IBD was defined by clinical, radiological, endoscopic and histological criteria. None of the study participants had taken antibiotics or used colon-cleansing products for at least 2 months prior to enrolment. Fecal samples were collected from 182 patients with IBD. Whole stools were collected in sterile boxes and immediately homogenized, and 0.2 g aliquots were frozen at −80°C for further analysis. Details of the genomic DNA extraction, 16S rRNA gene sequencing, and additional real-time quantitative PCR are presented in the **S1 Text**.

### 16S rRNA gene sequencing

Genomic DNA was extracted from 200 mg of feces as described previously[45]. Following microbial lysis involving both mechanical and chemical step, nucleic acids were precipitated by isopropanol for 10 minutes at room temperature, followed by incubation for 15 minutes on ice, and centrifugation for 30 minutes at 15,000g and 4°C. Pellets were suspended in 112 μL of phosphate buffer and 12 μL of potassium acetate. After the RNase treatment and DNA precipitation, nucleic acids were be recovered by centrifugation at 15,000g and 4°C for 30 minutes. The DNA pellet were suspended in 100 μL of TE buffer. After extraction, the total DNA concentration was measured using PicoGreen (Invitrogen), and global 16S gene DNA copy numbers were measured using a qPCR method adapted from Maeda et al[46] allowing for inhibition effect estimation and DNA concentration adjustment.

The sequence region of the 16S rRNA gene spanning the variable region V3-V5 was amplified using the broad-range forward primer For16S_519 (CAGCMGCCGCGGTAATAC) and reverse primer Rev16S_926 (CCGTCAATTCMTTTGAGTTT). Amplification reaction (initial activation step at 94°C for 1 min followed by 30 cycles of 94°C for 15 s, 43°C for 15 s and 68°C for 45 s plus final incubation at 68°C for 1 min) was performed in a total volume of 100 μL containing 1X PCR buffer, 2 mM MgSO_4_, 1 U of DNA High Fidelity Taq Polymerase (Invitrogen, Carlsbad, CA), 625 nM of each barcoded primer (IDT), 250 μM of each dNTP (Invitrogen) and the concentration-adjusted DNA sample. A bidirectional library was prepared using the One Touch2 Template Kit and sequenced on PGM Ion Torrent using the Ion PGM Sequencing 400 Kit (Life Technologies, Carlsbad, CA).

The sequences were demultiplexed and quality filtered using the Quantitative Insights Into Microbial Ecology (QIIME, version 1.8.0) software package. The sequences were trimmed for barcodes and PCR primers and were binned for a minimal sequence length of 200 pb. The sequences were then assigned to Operational Taxonomic Units (OTUs) using the UCLUST algorithm[47] with a 97% threshold pairwise identity and taxonomically classified using the Greengenes reference database[48]. Rarefaction was performed (10,000 and 2,000 sequences per sample respectively for discovery and replication cohorts). Rarefied reads counts were then transpose into per-individual proportion and used to compare OTUs abundances across samples. We identified a total of 897 bacterial taxa across 6 hierarchical levels (Phylum, Class, Order, Family, Genus and Species). However 577 taxa were detected in less than 20% of the participants (**S1 Fig**), and were therefore removed to limit sparsity issue in further analyses.

### Bacterial level pre-processing

Large-scale genomics data (e.g. microarray, proteomic, metabolomic) are commonly pre-process, applying various transformation and normalization procedures to remove confounding effects[49] but also to increase power[50]. Pre-processing can strongly impact both power and robustness[51] and should therefore be conducted carefully. To our knowledge, there is no established consensus on how to perform optimal pre-processing of microbiome data. The hierarchical relationship between taxa (**Fig 1**), along with strong pairwise correlation within each level (**S3 Fig**), makes pre-processing particularly challenging. Moreover, as we recently showed[52], standard approaches such as adjusting outcomes for the top principal components (PCs) of the taxa level (as commonly done in gene expression analysis[53]) can potentially introduce bias in our analysis–a phenomenon that would be amplified in our data because PCs can capture the shared variability of specific bacterial families. Therefore, we focused the pre-processing on transformation and filtering that will ensure the validity of the statistical tests. In particular, linear regression is sensitive to the presence of outliers, assumes a normal distribution of the outcome residual, and requires sufficient sample size for reliable effect estimation when multiple predictors are considered. The raw distributions of bacterial levels show widespread and strong outliers and skewed distribution.

In practice, we first filtered out all elements quantified in <20% of individuals or poorly annotated. We did not to use a more stringent threshold to allow for a screening as broad as possible, while we validated the most significant results through replication analysis in independent datasets. However, as showed in our sensitivity analyses (**S13 Fig**) we expect very low power to identify association with taxa quantified in less than 70% of the individuals in our dataset. Indeed, all top results from our assocaition analysis (**Table 2**) involve taxa were present in more than 85% of the individuals (e.g. *Fprau* was quantified in more than 95% of the individuals). Second, we applied rank-based inverse normal transformation to address both non-normal distribution of the bacteria levels and avoid false signal due to outliers. We acknowledge that such non-linear transformation can affect the inference [54]. However, rank-based inverse normal transformation is very commonly used in marginal genetic effect analyses of ‘omics data (e.g. microarray, RNA-seq, metabolites, which have properties similar to our microbiome data) and the current consensus is that, despite drawbacks, it remains a simple and efficient solution in many settings as compared to more complex approaches [55, 56]. Moreover, we also noted that rank-based inverse normal transformation is also now used in some microbiome data analysis[11, 57].

**Table 2:** Bacteria-genetic variant associations.

Outcome	Gene	Disease	Discovery		Replication		Meta-analysis	
			β*	*(Pval)*	β*	*(Pval)*	β*	*(Pval)*
*Firmicutes*	*CARD9*	IBD	-0.38	*(2*.*3x10*^*-4*^*)*	-0.08	*(0*.*50)*	-0.25	*(1*.*1x10*^*-3*^*)*
		CD	-0.43	*(7*.*4x10*^*-4*^*)*	-0.05	*(0*.*76)*	-0.30	*(2*.*8x10*^*-3*^*)*
		CDil	-0.44	*(2*.*7x10*^*-3*^*)*	-0.08	*(0*.*67)*	-0.31	*(6*.*4x10*^*-3*^*)*
		CDni	-0.46	*(0*.*23)*	0.85	*(0*.*041)*	0.12	*(0*.*66)*
		UC	-0.25	*(0*.*19)*	0.07	*(0*.*74)*	-0.10	*(0*.*46)*
*Bacteroides*	*NOD2*	IBD	-0.62	*(1*.*9x10*^*-4*^*)*	-0.05	*(0*.*58)*	-0.20	*(0*.*014)*
		CD	-0.57	*(4*.*1x10*^*-3*^*)*	-0.17	*(0*.*20)*	-0.30	*(6*.*3x10*^*-3*^*)*
		CDil	-0.65	*(3*.*3x10*^*-3*^*)*	-0.16	*(0*.*26)*	-0.31	*(8*.*7x10*^*-3*^*)*
		CDni	-		-0.42	*(0*.*41)*	-0.42	*(0*.*41)*
		UC	-0.50	*(0*.*18)*	0.24	*(0*.*15)*	0.11	*(0*.*47)*
*Roseburia*	*NOD2*	IBD	-0.58	*(6*.*4x10*^*-5*^*)*	-0.22	*(0*.*059)*	-0.36	*(4*.*9x10*^*-5*^*)*
		CD	-0.46	*(6*.*7x10*^*-3*^*)*	-0.26	*(0*.*076)*	-0.35	*(1*.*5x10*^*-3*^*)*
		CDil	-0.30	*(0*.*082)*	-0.34	*(0*.*030)*	-0.32	*(5*.*2x10*^*-3*^*)*
		CDni	-		-0.27	*(0*.*61)*	-0.27	*(0*.*61)*
		UC	-0.76	*(0*.*0052)*	0.14	*(0*.*54)*	-0.25	*(0*.*14)*
*F*. *prausnitzii*	*NOD2*	IBD	-0.56	*(4*.*0x10*^*-4*^*)*	-0.30	*(0*.*014)*	-0.40	*(3*.*2x10*^*-5*^*)*
		CD	-0.48	*(6*.*4x10*^*-3*^*)*	-0.20	*(0*.*19)*	-0.32	*(4*.*7x10*^*-3*^*)*
		CDil	-0.51	*(0*.*014)*	-0.31	*(0*.*050)*	-0.39	*(2*.*0x10*^*-3*^*)*
		CDni	-		-0.08	*(0*.*86)*	-0.08	*(0*.*86)*
		UC	-0.32	*(0*.*33)*	-0.14	*(0*.*61)*	-0.22	*(0*.*31)*

Abbreviation: CD, Crohn’s disease; UC, ulcerative colitis; CDil, CD ileal; CDni, CD non-ileal

^*^Beta coefficients were derived when using the allele associated with an increased risk of IBD as the coded allele. Outcomes were normalized. All outcomes had a variance of 1, whereas the genetic variants were not transformed; thus, the beta coefficients corresponded to the estimated change in the outcome mean per additional risk allele.

### Statistical analysis

We performed a systematic screening for association between the selected genetic variants and bacterial level. Association test were performed using standard univariate linear regression including seven potential confounding factors: gender, age, smoking, and treatment for 5-aminosalicylic acid, corticosteroids, anti-TNF agent, thiopurine or methotrexate. Importantly, we did not adjust the model for disease type (UC versus CD) as we demonstrated in a recent study that adjusting for variables that have a genetic basis might invalid association test [58]. To increase statistical power we also employed *CMS* (Covariates for Multiphenotype Studies), an approach recently developed by our group [19] to select additional covariates from the available bacterial data. Hence the final test we applied consisted in evaluating ${\hat{\beta }}_{G}$, the estimated effect of the genetic variant *G* on the quantification of a bacteria *Y*, in the following model:

$$Y∼\beta_{0}+\beta_{G}G+\beta_{Z}Z+\beta_{C}C$$

where **β**_**Z**_ and **β**_**C**_ are vectors of effect of **Z**, the counfounding factors, and **C**, a set of bacteria selected by *CMS*. To account for the hierarchical structure of the data, *CMS* search for covariates **C** of an outcome *Y* was conducted only within taxa of the same hierarchical levels as *Y*.

### Replication study

We performed a replication of our main results using two independent studies. Both studies are themselves combination of multiple cohorts. The first study included 474 adult IBD cases from three cohorts. We only considered two of them, MGH (Mass General Hospital, N = 170) and MSH (Mont Sinai Hospital, N = 65), both from the United States. We discarded the last one because of missing data on the main confounding factors. In these two cohorts mucosa associated microbiota was analyzed from intestinal biopsies. Because of major differences in the composition and treatment between MGH and MSH (**S4 Table**), we analyzed the two cohorts separately and refer to them as *replication 1* and *replication 2*. Moreover, all participants that undergone proctocolectomy with ileoanal anastomosis were excluded *a priori*, as the microbiota composition might be dramatically impacted by this operation and therefore not reflect the overall population. The second study included 216 IBD cases from a Netherland cohort with almost complete covariates data [21]. We refer to this dataset as *replication 3*. All replication analyses (1, 2, and 3) were adjusted for gender, age, smoking, mesalamine, antibiotics, and immunosuppressant treatments. To ensure our discovery signal was not biased because of the used of covariates identified by the *CMS* approach, we did not use *CMS* at the replication stage. Finally, we performed an inverse-variance meta-analysis of all three replications dataset using the *R metafor* package. Study-specific results are presented in **S5 Table**.

### Evaluation of ascertainment bias

The established association between IBD and both the genetic variants and the bacterial levels implies that the joint distribution of these variables in IBD cases only might not representative of the overall population. This is a known issue that has been widely discussed in the context of secondary trait analysis [28, 29, 59–62]. Indeed, with the surge of case-control genome-wide association study, a number of investigators are facing the problem of analyzing secondary phenotypes, measured on behalf of the disease status, while accounting for the sample ascertainment. These works show that when mishandled, secondary trait analysis can be biased. In particular, if a predictor *X* (here, a genetic variant) and a secondary phenotype *Y* (here, a bacterial level) are associated with the outcome (here, IBD), the two variables might display a false association. While studies showed that for rare diseases (*i*.*e*. prevalence ≤ 1%, as for IBD), and small effect, the *Y*~*X* association test in disease cases only, as done in this study, is expected to be small [28]. Estimating the changes in expected effect estimates depending on sampling design is a non-trivial problem. As discussed in the literature for logistic models [29, 63],it has a direct relationship with the parametrization of the underlying generating models.

Consider a biallelic genetic variant *g*, generated from a binomial distribution with minor allele frequency *p*, a bacterial level *b* following a distribution Ω and a disease status *d* with probability *π*. For mathematical convenience we further consider *D*, *B* and *G* for standardized *d*, *b* and *g*, respectively. We are interested in estimating Δ = *β*_*d* = 1_−*β*, the difference between the regression coefficient between *G* and *B* in the general population (*β*). Without assuming any particular distribution of the variables, Δ has the general form:

$$\begin{array}{l} \Delta \;=\beta_{d=1}-\beta \\ \;=\frac{cov(B,G|d=1)}{\sigma_{G|d=1}^{2}}-\frac{cov(B,G)}{\sigma_{G}^{2}}\\ \;=\frac{E[BG|d=1]-E[B|d=1]E[G|d=1]}{\sigma_{G|d=1}^{2}}-E[BG] \end{array}$$

where $\sigma_{G|d=1}^{2}=var(G|d=1)$ is the variance of *G* conditional on *d* = 1. As E[B]=E[G]=E[D]=0, the expectation conditional on *d* can we be rewritten as:

$$\begin{array}{l} E[BG|d=1]=E\left[BG|D=\frac{\left(1-\mu_{d}\right)}{\sigma_{d}}\right]\\ \;=E[BGD]\frac{\left(1-\mu_{d}\right)}{\sigma_{d}}+E[BG]\\ \;=E[BGD]\frac{\sigma_{d}}{\mu_{d}}+E[BG]\\ E[B|d=1]=E\left[B|D=\frac{\left(1-\mu_{d}\right)}{\sigma_{d}}\right]\\ \;=E[BD]\frac{\sigma_{d}}{\mu_{d}}\\ E[G|d=1]=E\left[G|D=\frac{\left(1-\mu_{d}\right)}{\sigma_{d}}\right]\\ \;=E[GD]\frac{\sigma_{d}}{\mu_{d}}\\ E[G^{2}|d=1]=E\left[G^{2}|D=\frac{\left(1-\mu_{d}\right)}{\sigma_{d}}\right]\\ \;=E[G^{2}D]\frac{\sigma_{d}}{\mu_{d}}+E[G^{2}]\\ \;=E[G^{2}D]\frac{\sigma_{d}}{\mu_{d}}+1\\ \sigma_{G|d=1}^{2}\;=E[{\left(G-E[G|d=1]\right)}^{2}|d=1]\\ \;=E[G^{2}-2GE[G|d=1]+{E[G|d=1]}^{2}|d=1]\\ \;=E[G^{2}|d=1]-{E[G|d=1]}^{2}\\ \;=1+E[G^{2}D]\frac{\sigma_{d}}{\mu_{d}}-{\left(E[GD]\frac{\sigma_{d}}{\mu_{d}}\right)}^{2} \end{array}$$

So without loss of generality, the bias Δ, equals:

$$\begin{array}{l} \Delta \;=\frac{E[BGD]\frac{\sigma_{d}}{\mu_{d}}+E[BG]-E[GD]E[BD]{\left(\frac{\sigma_{d}}{\mu_{d}}\right)}^{2}}{\sigma_{G|d=1}^{2}}-E[BG]\\ \;=\frac{E[BGD]\frac{\sigma_{d}}{\mu_{d}}-E[GD]E[BD]{\left(\frac{\sigma_{d}}{\mu_{d}}\right)}^{2}}{\sigma_{G|d=1}^{2}}+E[BG]\frac{1-\sigma_{G|d=1}^{2}}{\sigma_{G|d=1}^{2}} \end{array}$$

Note that the above formulation of Δ is valid for any generating model and data distribution.

### Model fit using estimates of selection bias

We are interested in assessing the impact of the bias Δ on the SNP-bacteria association for the four hypothetical causal models (**Fig 3A–3D**) in order to determine their fitness to our data. Here we assumed IBD status follows a binomial distribution with *n* = 1 and is linked to other variable through a logistic model, and we constrained the model to fit known associations: the positive association between the genetic variants and IBD [3, 4], and the negative association between the four taxa (*Roseburia* genus, *Bacteroidia* class, *F*. *prausnitzii*, and *Firmicutes* phylum) and IBD [8, 24, 64]. In model a) the effect of *g* on *D* is mediated by *B*. In model b) the effect of *g* on *B* is mediated by *D*. In model c), *g* and *B* act independently on *IBD* and are not associated in the population. Finally in model d), *B* and *D* are both influenced by *U*, the unmeasured risk factors.

The data can be expressed through the following generative models:

$$\begin{array}{l} \mathrm{Model}\;a):\;\left\{ \begin{array}{l} B=\beta G+\varepsilon_{B}\\ E[d|B]=1/\left[1+e^{-(\omega_{0}+\omega_{B}B)}\right] \end{array}\right.\\ \mathrm{Model}\;b):\;\left\{ \begin{array}{l} B=\beta G+\varepsilon_{B}\\ E[d|G]=1/\left[1+e^{-(\omega_{0}+\omega_{G}G)}\right] \end{array}\right.\\ \mathrm{Model}\;c):\;\{E[d|B,G]=1/\left[1+e^{-(\omega_{0}+\omega_{B}B+\omega_{G}G)}\right]\\ \mathrm{Model}\;d):\;\left\{ \begin{array}{l} B=\gamma D+\varepsilon_{B}\\ E[d|G]=1/\left[1+e^{-(\omega_{0}+\omega_{G}G)}\right] \end{array}\right. \end{array}$$

Building on our estimator of Δ and leveraging previous work relating logistic and linear model [63, 65] we further demonstrate that when the disease is common, under the assumption of normality of *B* and *G* and small effects of the independent variables, Δ for the four hypothetical causal models can be approximated as (**S1 Text** and **S8 Fig**):

$$\begin{array}{l} \Delta_{a}\approx -\beta \frac{\omega_{B}^{2}{\left(1-\mu_{d}\right)}^{2}(1-\beta^{2})}{\sigma_{G|d=1}^{2}}\\ \Delta_{b}\approx 0\\ \Delta_{c}\approx -\omega_{G}\omega_{B}\frac{{\left(1-\mu_{d}\right)}^{2}}{\sigma_{G|d=1}^{2}}\\ \Delta_{d}=E[BG]\approx -\gamma \omega_{G}\sigma_{d} \end{array}$$

However, the normality assumption will not hold in general for both *G* (being drawn from a binomial) and *B* (e.g. drawn from a negative binomial), and the two other hypothesis (common disease and small effect size) are also violated in our data. Nevertheless, as previously showed [65], and as confirmed by our simulations (**S1 Text** and **S9–S12 Figs**), parameters from the linear approximation have the proper sign, so that the above Δ approximations is still informative about the direction of the bias, whatever the residual distribution of *B* and *G*. It follows that:

$$\begin{array}{l} sign(\Delta_{a})=-sign(\beta )\\ sign(\Delta_{c})=-sign(\omega_{G}\omega_{B})\\ sign(\Delta_{d})=-sign(\gamma \omega_{G}) \end{array}$$

Thus, in *model a)* the effect estimate will be biased toward the null, *i*.*e* the magnitude of the genetic effect on bacteria will be underestimated. In *model b)*, we expect the effect to be the same in case only sample as in the whole population. In *model c)*, the ascertainment will induce a bias which direction has opposite sign to the product of the genetic effect on IBD and the bacterium effect on IBD. Finally In *model d)*, we expect the indirect effect of the genetic variant on the bacterium to be null in a case only sample.

## Supporting information

S1 Text Supplementary methods. (DOCX)

S1 Table Characteristics of the genetic variants analyzed. (DOCX)

S2 Table Significant association within each hierarchical level. (DOCX)

S3 Table Type I error rate under four hypothetical models. (DOCX)

S4 Table Characteristics of the replication studies. (DOCX)

S5 Table Replication analysis per cohort. (DOCX)

S1 Fig Non-zero abundance of taxa. Density (a) and cumulative distribution (b) of the percentage of non-zero abundance for each of 897 bacterial taxa across the 182 IBD cases analyzed. The red and blue dashed lines in (b) correspond to a non-zero abundance of 95% and 80%, respectively. There was 494 (~55%) and 320 taxa (~36%) with lower values, respectively, *i*.*e*. taxa that are present in more than 5% and 20% of the participants, respectively.
(TIF)

S2 Fig Overview of the bacterial taxa distribution in IBD controls. Relative proportion of each of the 168 bacterial taxa analyzed across each of the six hierarchical levels. Only taxa representing more than 0.5% of the total bacterial loading are labelled. Grey areas correspond to unknown, unmeasured, or underrepresented taxa.
(TIF)

S3 Fig Pairwise correlation between bacterial level. Pairwise Pearson correlation between bacterial taxa derived across the 182 IBD cases. Taxa were grouped by hierarchical strata (Phylum, Class, Order, Family, Genus and Species), so that the panels from the diagonal represent the correlation within each stratum, while off-diagonal panels present cross-strata correlation. Strength of correlation is presented as a gradient from dark blue (-1) to dark red (1).
(TIF)

S4 Fig Summary of association signals for ATG16L1. Bacterial levels were tested for association with SNP rs12994997 from gene ATG16L1. Positive and negative associations are represented as gradient of orange and blue, respectively. The horizontal axis indicates the corresponding–log_10_(p-value). Results are presented across the taxa hierarchy. Empty cells indicate that the subsequent element is unknown or unmeasured in our samples.
(TIF)

S5 Fig Summary of association signals for CARD9. Bacterial levels were tested for association with SNP rs10781499 from gene CARD9. Positive and negative correlations are represented as gradient of orange and blue, respectively. The horizontal axis indicates the corresponding–log_10_(p-value). Results are presented across the taxa hierarchy. Empty cells indicate that the subsequent element is unknown or unmeasured in our samples.
(TIF)

S6 Fig Summary of association signals for LRRK2. Bacterial levels were tested for association with SNP rs11564258 from gene LRRK2. Positive and negative correlations are represented as gradient of orange and blue, respectively. The horizontal axis indicates the corresponding–log_10_(p-value). Results are presented across the taxa hierarchy. Empty cells indicate that the subsequent element is unknown or unmeasured in our samples.
(TIF)

S7 Fig Summary of association signals for NOD2. Bacterial levels were tested for association with the genetic risk score of gene NOD2 (rs2066844 + rs2066845 + rs2066847). Positive and negative correlations are represented as gradient of orange and blue, respectively. The horizontal axis indicates the corresponding–log_10_(p-value). Results are presented across the taxa hierarchy. Empty cells indicate that the subsequent element is unknown or unmeasured in our samples.
(TIF)

S8 FigLinear approximation of logistic model.We simulated four datasets including each 20,000 individuals. For each dataset we draw a genetic variant *G* from a binomial assuming a minor allele frequency of 0.1, a bacterial level *B* while using arbitrarily various distributions, and a disease status *d* generated from a logistic model, $E[d|B,G]=1/\left(1+e^{-[\omega_{0}+\omega_{B}B+\omega_{G}G]}\right)$. We then performed linear regression of the disease probability while including an increasing number of polynomial terms of the predictors *B* and *G* and their interactions (*model0* to *model3*). At the two extremes, m*odel0* includes only the marginal effects of the predictor ($E[d|B,G]∼\lambda_{0}+\lambda_{G}G+\lambda_{B}B$), while *model3* include polynomial up to a power of 4 and any relevant interactions. For each model we plotted the simulated disease probability against the fitted values for *model0* to *model3*. We use four sets of parameters to illustrate the requirement for additional terms in the linear model as the prevalence moves away from 0.5 and effect are getting larger. In (a) we considered a disease prevalence of 0.5, a normally distributed *B*, and modest *B* and *G* effects. In (b) we slightly increase effects and considered a rare disease case. In (c) we used generated *B* from an exponential, consider low disease prevalence and large effects. Finally, in (d) we considered a rare disease, very large effects and generated *B* from a uniform distribution.(TIF)

S9 FigBias for common disease and large effect.We simulated series of 1,000 replicates each including 100,000 individuals. For each replicate we draw a genetic variant *G*, a bacterial level *B*, and a disease status *d* using the two equations *B* = *βG* + *γD* + *ε*_*B*_ and $E[d|B,G]=1/\left(1+e^{-[\omega_{0}+\omega_{B}B+\omega_{G}G]}\right)$. We use three sets of parameters to match model *a* (left column), model *b* (middle column) and models *c* and *d* (right column), while changing the direction of the effect. In this simulation, all effects (panel a) were assumed to be large and disease prevalence high (~30%). For each simulation we estimated through standard linear regression the association coefficient between *B* and *G* in the whole population and in the cases only. Median of the coefficient in the whole population is indicated by a bold red line, while coefficients observed in cases only are provided in boxplots. The difference between the two coefficients (case only—whole population), Δ, is indicated in blue if negative and in pink if positive. For each set of parameters (panel a), we derived the proposed approximation of Δ (panel b), and the aforementioned estimates while drawing *ε*_*B*_ from a normal distribution (panel c), an exponential distribution (panel d), and a uniform distribution (panel e).(TIF)

S10 FigBias for common disease and moderate effect.We simulated series of 1,000 replicates each including 100,000 individuals. For each replicate we draw a genetic variant *G*, a bacterial level *B*, and a disease status *d* using the two equations *B* = *βG* + *γD* + *ε*_*B*_ and $E[d|B,G]=1/\left(1+e^{-[\omega_{0}+\omega_{B}B+\omega_{G}G]}\right)$. We use three sets of parameters to match model *a* (left column), model *b* (middle column) and models *c* and *d* (right column), while changing the direction of the effect. In this simulation, all effects (panel a) were assumed to be large and disease prevalence high (~30%). For each simulation we estimated through standard linear regression the association coefficient between *B* and *G* in the whole population and in the cases only. Median of the coefficient in the whole population is indicated by a bold red line, while coefficients observed in cases only are provided in boxplots. The difference between the two coefficients (case only—whole population), Δ, is indicated in blue if negative and in pink if positive. For each set of parameters (panel a), we derived the proposed approximation of Δ (panel b), and the aforementioned estimates while drawing *ε*_*B*_ from a normal distribution (panel c), an exponential distribution (panel d), and a uniform distribution (panel e).(TIF)

S11 FigBias for rare disease and large effect.We simulated series of 1,000 replicates each including 100,000 individuals. For each replicate we draw a genetic variant *G*, a bacterial level *B*, and a disease status *d* using the two equations *B* = *βG* + *γD* + *ε*_*B*_ and $E[d|B,G]=1/\left(1+e^{-[\omega_{0}+\omega_{B}B+\omega_{G}G]}\right)$. We use three sets of parameters to match model *a* (left column), model *b* (middle column) and models *c* and *d* (right column), while changing the direction of the effect. In this simulation, all effects (panel a) were assumed to be large and disease prevalence high (~30%). For each simulation we estimated through standard linear regression the association coefficient between *B* and *G* in the whole population and in the cases only. Median of the coefficient in the whole population is indicated by a bold red line, while coefficients observed in cases only are provided in boxplots. The difference between the two coefficients (case only—whole population), Δ, is indicated in blue if negative and in pink if positive. For each set of parameters (panel a), we derived the proposed approximation of Δ (panel b), and the aforementioned estimates while drawing *ε*_*B*_ from a normal distribution (panel c), an exponential distribution (panel d), and a uniform distribution (panel e).(TIF)

S12 FigBias for rare disease and moderate effect.We simulated series of 1,000 replicates each including 100,000 individuals. For each replicate we draw a genetic variant *G*, a bacterial level *B*, and a disease status *d* using the two equations *B* = *βG* + *γD* + *ε*_*B*_ and $E[d|B,G]=1/\left(1+e^{-[\omega_{0}+\omega_{B}B+\omega_{G}G]}\right)$. We use three sets of parameters to match model *a* (left column), model *b* (middle column) and models *c* and *d* (right column), while changing the direction of the effect. In this simulation, all effects (panel a) were assumed to be large and disease prevalence high (~30%). For each simulation we estimated through standard linear regression the association coefficient between *B* and *G* in the whole population and in the cases only. Median of the coefficient in the whole population is indicated by a bold red line, while coefficients observed in cases only are provided in boxplots. The difference between the two coefficients (case only—whole population), Δ, is indicated in blue if negative and in pink if positive. For each set of parameters (panel a), we derived the proposed approximation of Δ (panel b), and the aforementioned estimates while drawing *ε*_*B*_ from a normal distribution (panel c), an exponential distribution (panel d), and a uniform distribution (panel e).(TIF)

S13 Fig NOD2-bacteria association in all IBD cases and CD cases only. We compared effect estimates for NOD2-bacteria association derived using standard linear regression, after adjusting for confounding factors, in all IBD cases (*β*_*IBD*_) and CD cases only (*β*_*CD*_). The gradient of colors (pink to red) and size of each point (small to large) indicate increasing significance of NOD2-bacteria association in the complete IBD cases dataset. Under the confounding model (b), where CD-bacteria association is partly confounded by a shared genetic effect of NOD2 on both outcomes, the two estimates have the same expectation and therefore the regression slope between the two estimates should be equal to 1 (black line). The observed slope (red line), which equals 0.88, is significantly different from 1 (*P* = 5e-3, tested using a student *t*-test assuming an expected slope equals to 1).
(TIF)

S14 Fig Impact of data sparsity. We simulated series of 20,000 replicates each including 10,000 individuals. For each individual, we generated a genetic variant, a bacteria and a case-control status following the four models (a, b, c and d) from **Fig 3**. The genotype was simulated using a binomial distribution with frequency of the coded allele randomly drawn in [0.05, 0.95]. The bacterial level was simulated using a negative binomial distribution were the dispersion parameters of each replicate were chosen so that the proportion of zero value vary from 0% to 95% across replicates. The case-control status was drawn from a binomial distribution with probability derived using a logit function with parameters matching the each of the four models. For each replicate, a subset of 200 cases was randomly chosen and used to test for association between the genotype and the bacteria using standard linear regression. The left (a) and middle (b) panels show the signed explained variance obtain from this experiment before and after applying an inverse-rank based normal transformation of the bacterial level, respectively. The red line shows the trend derived using local fitting as implemented in R loess function with default parameters. The right panels (c) present median *p*-values from the latter experiment derived over bins of replicates with a range of percentage of zero-values.
(TIF)

S15 Fig Impact of effect heterogeneity on bias. We simulated series of 10,000 replicates each including 30,000 individuals. For each individual, we generated a genetic variant *G*, a bacteria *B* and two case-control status, CD, which was defined based on the four causal models (a, b, c and d) from **Fig 3**, and UC, which was drawn independently of other variables. The two diseases were merged to form the IBD status. The genotype was simulated using a binomial distribution with frequency of the risk allele randomly drawn in [0.05, 0.2]. The bacterial level was simulated using a negative binomial distribution while randomly drawing the dispersion parameter so that the proportion of zero-value ranges in [0%, 80%]. The case-control status was drawn from a binomial distribution with probability derived using a logit function. All parameters from the simulation were set so that it match estimates from the literature and our primary results. The three lower panels show empirical distribution of those parameters estimated in the whole population. Disease prevalence, genetic effect and bacteria-disease association matched both in direction and magnitude for all causal models. We then randomly sampled from each replicate, a subset of 200 cases tested for association between *G* and *B* using standard linear regression, and after applying an inverse rank-based normal transformation of *B*. The test was applied in the sub-sample of IBD cases, but also in the sub-group of CD cases only and the sub-group of UC cases only.
(TIF)

S16 Fig Assuming an intermediate variable confounds the effect on IBD. We performed a simulation similar to Fig 3, expect that we replaced IBD status by a severity score. We plotted regression slopes between bacteria and the genetic variant in the whole population, and in cases only, using either the raw bacterial data or after adjusting for severity. Top panels (a, b, c, and d) present the hypothetical causal diagrams and bottom panels (e, f, g, and h) present the corresponding scatterplots of *B* as a function of *g* in the population (dark grey points, and trend in black), in cases only (light grey points and trend in red), and again, in cases only but after adjusting the bacteria for severity (blue crosses, and trend in dark blue). In model a) the effect of *g* on severity is mediated by *B*; in cases the effect of *g* on *B* is underestimated because of the oversampling of participants carrying risk alleles (e). In model b) the genetic variant influences both severity and *B*, inducing a correlation between severity and *B* which is observed in both the whole sample and cases only (f). In model c), *g* and *B* act independently on severity and are therefore not associated in the population, however *g* and *B* are positively correlated in case-only samples because of biased selection (g). Finally, in model d) the effect of *g* on *B* is mediated by severity; the indirect association between *g* and *B* observed in the general population is still present in cases only, but is canceled when adjusting bacteria for severity (h).
(TIF)

S17 FigSensitivity analysis adjusting for flare-remission.We performed the same analysis as for Fig 4, expect that we adjusted all analyses for flare-remission. The 168 bacterial taxa were tested for association with the variants from each of the four genes considered: (a) ATG16L1 (rs12994997), (b) CARD9 (rs10781499), (c) LRRK2 (rs11564258), and (d) NOD2 (rs2066844, rs2066845, and rs2066847). The histograms on the left panel show the distribution of IBD risk alleles-bacteria association (i.e. of ${\hat{\beta }}_{g}$, the regression coefficients) and the enrichment for negative effects (in blue, p-values equal 0.018, 1.3x10-8, 9.3x10-6, 0.018, respectively). Panel (e) shows a similar histogram while merging the per-risk allele change in bacteria level of the four genes (i.e. summing for each bacteria the ${\hat{\beta }}_{g}$ of the four genes). Panel (f) shows the distribution of bacteria-IBD association derived in an IBD cases-controls dataset (${\hat{\beta }}_{B}$) for each bin from panel (e). Together, panels (e) and (f) show the strong concordance of the gene-bacteria and bacteria-IBD effects, in agreement with a mediation effect of the risk allele on IBD through the microbiome. In particular, bacteria displaying lower level in carrier of IBD risk alleles are more likely to be negatively associated with the risk of IBD.(TIF)
