# Supplementary material for: Genetic effects on the commensal microbiota in inflammatory bowel disease patients
Source: PLoS Genet. 2019 Mar 8;15(3):e1008018. doi: 10.1371/journal.pgen.1008018 (PMC6426259; doi:10.1371/journal.pgen.1008018)
Supplement: S1 Text — (DOCX) [file pgen.1008018.s001.docx]

**S1 text. Supplementary Methods**

Table of Contents

[Real-time quantitative PCR 2](#_Toc1470921)

[Genotyping and quality control 2](#_Toc1470922)

[Covariate selection by CMS 2](#_Toc1470923)

[Simulation study 2](#_Toc1470924)

[Potential limitation 3](#_Toc1470925)

[Re-parametrization of the logistic model with a linear probability function 4](#_Toc1470926)

[Without the linear approximation of the Logistic model 8](#_Toc1470927)

[Alternative study design 9](#_Toc1470928)

# Real-time quantitative PCR

As 16S sequencing approach is known to have some limitation regarding the accuracy of the quantification [23], we performed a quantitative analysis of *F. prausnitzii* on fecal DNA extracted from weighted human stool samples via real-time quantitative PCR using an ABI 7000 Sequence Detection System apparatus with 7000 system software v. 1.2.3 (Applied Biosystems, Foster City, CA, USA). Amplification and detection were carried out in 96-well plates and with Takyon^TM^ SYBR Green PCR kit (Eurogentec, Liege, Belgium). The probes and primers for the bacterial 16S rRNA genes described previously were used [22]. The threshold cycle for each sample was determined for each gene normalized to the C_T_ value of the all-bacteria 16S ribosomal RNA gene. Data were calculated using the 2^-ΔΔCt^ method [45].

# Genotyping and quality control

Patients with IBD were genotyped using Fluidigm technology (UMR CNRS 8199, Lille, France) for the following SNPs from five genes: rs12994997 (*ATG16L1*, risk allele A), rs10781499 (*CARD9*, risk allele A), rs11564258 (*LRRK2*, risk allele A), rs11209026 (*IL23R*), and three *NOD2* variants, rs2066847 (insertion), rs2066845 (risk allele C), and rs2066844 (risk allele T). The characteristics of the variants are presented in **S1 Table.** However, the very low frequency of the *IL23R* variant (minor allele frequency of 0.028) prevented from performing a valid association test, and was therefore removed from the analysis. The variants from *ATG16L1*, *CARD9*, and *LRRK2* were coded assuming additive effects of the risk allele. The *NOD2* variants had low to very low frequencies (**S1 Table**) and were therefore aggregated into a binary variable defined as the presence/absence of at least one allele associated with increased risk of IBD: SNPs rs2066844 (C > T), rs2066845 (G > C) and rs2066847 (insertion).

# Covariate selection by CMS

*CMS*(Covariates for Multiphenotype Studies) is an approach recently developed by our group for the analysis of multivariate dataset [19]. It aims that identify for each specific univariate test between a predictor and a primary outcome, a subset of variables from the data that can be used as proxy for explanatory variable of the primary outcome. Once selected, these variables can simply be included in the model as covariates. In brief, for each univariate test between a genetic variant and a given bacterium (the primary outcome), the *CMS* approach parses other bacterial levels correlated to the primary outcome and identifies those that can be added as covariate in the regression model without introducing bias [58]. More precisely, the *CMS* targets bacteria that likely vary with the primary outcome because of shared explanatory variables, and can therefore be used as proxy for unmeasured causal exposure(s) of the primary outcome. Including these bacteria as covariate in the regression model can reduce the primary outcome residual variance and increase power.

# Simulation study

We performed simulation studies for the four causal models across a wide range of scenarios to confirm the validity of our inference on effect estimate bias, and explore its robustness. In brief, we used a general framework where data were drawn based on the two following generating functions:

$$B=\beta G+\gamma D+\varepsilon_{B}$$

$$\mathbb{E}[d|B,G]=\frac{e^{\omega_{0}+\omega_{B}B+\omega_{G}G}}{1+e^{\omega_{0}+\omega_{B}B+\omega_{G}G}}$$

where parameters ($\beta,\gamma,\omega_{B},\omega_{G}$) were specifically set to 0 to match the desired model, i.e. $\omega_{G}=\gamma=0$ in model a); $\omega_{B}=\beta=0$ in model b); and $\beta=\gamma=0$ in model c) and d), while $\omega_{0}$, the baseline risk was set to match the desire disease prevalence. Except when stated otherwise, the genotype $G$ was drawn from a binomial distribution with minor allele frequency of 0.1. When influenced by the genotype (model a) or the disease (model b), the bacterium $B$ was simulated using the above standard linear model while considering various distribution for the residual $\varepsilon_{B}$. However, we also considered simulating $B$ using a negative binomial distribution using the following model

$$B=NB(\mu,\mu\left( 1+\mu\Phi\right))$$

where $\Phi$ is the dispersion parameter, and $\mu$, the expected value of $B$ is defined as $\mu=e^{\gamma_{0}+\gamma_{1}D+\gamma_{2}G+\gamma_{3}U}$, and $\gamma_{1}$ and $\gamma_{2}$ are set to zero depending on the scenario considered. Additional details on the simulation study are provided in the **SI Material and Methods**.

# Potential limitation

How to optimize microbiome data pre-processing is challenging. Empirical bacterial levels follow non-negative, over-dispersed distribution, and often harbor a large number of zeros values. It has been suggested to use Poisson distribution, negative binomial distributions, or hurdle models to analyze microbiome data [31]. Moreover, we applied a rank-based inverse-normal transformation in our real data analysis, while there remains sparsity (after QC, bacteria had a minimum of 20% of non-zero values). To circumvent the impact of data sparsity and data transformation, we performed a stringent filtering, removing all taxa quantified in less than 20% of the individuals. We did not to use a more stringent threshold to allow for a screening as broad as possible, while we validated the most significant results through replication analysis in independent datasets. We also applied rank-based inverse normal transformation to address both non-normal distribution of the bacteria levels and avoid false signal due to outliers. We acknowledge that such non-linear transformation can affect the inference [54]. However, rank-based inverse normal transformation is very commonly used in marginal genetic effect analyses of ‘omics data (e.g. microarray, RNA-seq, metabolites, which have properties similar to our microbiome data) and the current consensus is that, despite drawbacks, it remains a simple and efficient solution in many settings as compared to more complex approaches [55, 56]. Moreover, we also noted that rank-based inverse normal transformation is also now used in some microbiome data analysis [11, 57].

To assess the impact of the aforementioned pre-processing, we performed a simulation study where bacterial levels were drawn from a negative binomial distribution keeping all parameters fixed except the dispersion parameter in order to achieve between 0% and 95% of zero-value. For each replicate, SNP-bacteria effect was estimated using standard linear regression before and after applying the rank-based inverse-normal transformation as in the real data analysis. As showed in **S14 Fig**, which presents the results from this simulation for the four scenarios from **Fig 3a-d**, the only expected impact is a very low power to identify association with taxa quantified in less than 70% of the individuals in our dataset. However, all taxa from our primary results presented in **Table 2** were present in more than 85% of the individuals (e.g. *Fprau* was quantified in more than 95% of the individuals).

Another limitation is that we assumed all effects are homogeneous in our causal inference framework, i.e. Crohn’s disease and ulcerative colitis cases were treated as a single outcome variable (IBD). However, it is well appreciated that *NOD2* variants and *F. prausnitzii* are mostly associated with CD, and ileal CD in particular, but show little evidence for association with UC. To assess the impact of genetic heterogeneity, we performed a second simulation mimicking as closely as possible the real data. Here, SNP-disease and bacteria-disease associations were present only for a subset of the cases. As showed in **S15 Fig**, in this scenario, some of the bias from model (c) and (d) illustrated in **Fig 3g-h** decreased toward the null. Our simulations also show that under model (c), and (d) we expect no association signal in neither disease subtype strata nor in the whole IBD-cases sample.

Finally, future studies with improved study design might help deciphering further the observed signal. The heterogeneity of treatment across individuals and the availabilty of covariates informations across studies can bias the results of both association screening and causal inference. Previous works already highlighted the strong effect of many drug treatment and environmental exposures on the microbiota composition. To limit the impact of these factors, we focused our discovery and replication analyses on individuals which had data on major confounding factors. Future work might instead explore imputation methods to infer missing covariates, or performed stratified analysis in more homogenous sample. Also, we merged all three *NOD2* variants into a binary variable defined as the presence/absence of at least one variant. While the effect of each of these variants is established, their combined effect is not well defined [41-43]. Understanding the relative contribution of each *NOD2* variant to the overall signal we observed is of primary interest but would require larger sample size.

# Re-parametrization of the logistic model with a linear probability function

Consider a biallelic genetic variant$g$, generated from a binomial distribution with minor allele frequency $p$, a bacterial level $b$ following a distribution $\Omega$ and a disease status$d$ with probability $\pi$. For mathematical convenience we further consider $D$, $B$ and $G$ for standardized $d$, $b$ and $g$, respectively. In order to match standard modelization, we consider further the case where the effects of predictors on the disease status are drawn from a logistic function (but other link function, such as the liability threshold model might be explored), and the effects on $B$ are drawn from a linear model. Our four scenarios of interest can therefore be expressed with the two generating functions:

$$B=\beta G+\gamma D+\varepsilon_{B}$$

$$\mathbb{E}[d|B,G]=\frac{e^{\omega_{0}+\omega_{B}B+\omega_{G}G}}{1+e^{\omega_{0}+\omega_{B}B+\omega_{G}G}}$$

where parameters ($\beta,\gamma,\omega_{B},\omega_{G}$) can specifically be set to 0 to match the desired model, i.e. $\omega_{G}=\gamma=0$ in model a); $\omega_{B}=\beta=0$ in model b); and $\beta=\gamma=0$ in model c) and d), while $\omega_{0}$, the baseline risk is set to match the desire disease prevalence.

Solving $\Delta$ for the logistic function faces numerous challenges (see next section). Instead, we suggest the direction of the bias (i.e. the sign of $\Delta$) can easily be inferred by approximating the logistic function by a linear models. When the disease is neither rare nor too common (e.g. prevalence in 0.3-0.7), and main $G$ and $B$ effects are small, the log odds are almost a linear function of the probability, so that a simple marginal model of the predictors would approximate the logistic model very well (**S8a Fig**). However, for more general cases (i.e. low or large prevalence, or large effects), fitting the probability of the disease requires adding polynomial terms of the predictors and their interactions. For illustration purposes we plotted in **S8 Fig** simulated disease probability (from a logistic model) against the predicted values from linear probability models while increasing the model complexity, i.e. increasing $K$ in the following $\mathbb{E}\left[ Pr(d=1)\left| B,G \right. \right]\sim\sum_{K}^{i=0} \sum_{K}^{j=0} \lambda_{ij}B^{i}G^{i}-1$. Importantly, the additional parameters capturing the non-linear effects of $B$ and $G$ are only of interest for prediction purposes of limited interest if the goal is causal inference.

Moreover, previous research that aimed at estimating log odds from linear models showed that for a predictor $X$, under the assumption of normality of error of $X$, the parameter from the linear model can be approximated as $\sigma_{d}^{2}\omega_{X}$, where $\sigma_{d}^{2}$ and $\omega_{X}$ are the variance of the disease and the log odds, respectively [63, 65]. It follows that under all the above assumptions (disease prevalence around 0.5, small effect, and normality of errors), the following expected values can be approximated as:

$\mathbb{E}\left[ D | G \right]\approx\sigma_{d}\omega_{G}G$ $\mathbb{E}\left[ D | B \right]\approx\sigma_{d}\omega_{B}B$

$$\mathbb{E}\left[ d | B,G \right]\approx\sigma_{d}\omega_{B}B+\sigma_{d}\omega_{G}G+\mu_{d}$$

$$\mathbb{E}\left[ D|G,B \right]\approx\mathbb{E}\left[ \frac{d-\mu_{d}}{\sigma_{d}}|G,B \right]$$

$$\approx\sigma_{d}\left( \omega_{B}B+\omega_{G}G \right)$$

$$\mathbb{E}\left[ BGD \right]\approx\mathbb{E}\left[ BG \mathbb{E}\left[ D|G,B \right] \right]$$

$$\approx\sigma_{d}\mathbb{E}\left[ GB \left( \omega_{B}B+\omega_{G}G \right) \right]$$

$$\approx\sigma_{d}\left( \omega_{B}\mathbb{E}\left[ GB^{2} \right]+\omega_{G}\mathbb{E}\left[ G^{2}B \right] \right)$$

When applied to our causal models it results in the following approximations:

For model *a)*, $\omega_{G}=\gamma=0$ and $\mathbb{E}\left[ BG \right]=\beta$:

$$\mathbb{E}\left[ BGD \right] \approx\sigma_{d}\mathbb{E}\left[ BG\left( \omega_{B}B \right) \right]$$

$$\approx\omega_{B}\sigma_{d}\mathbb{E}\left[ G\left( \beta G+\varepsilon_{B} \right)^{2} \right]$$

$$\approx\omega_{B}\sigma_{d}\mathbb{E}\left[ \beta^{2}G^{3}+2\beta G^{2}\varepsilon_{B}+\varepsilon_{B}^{2}G \right]$$

$$\approx\omega_{B}\beta^{2}\sigma_{d}\mathbb{E}\left[ G^{3} \right]$$

$$\mathbb{E}\left[ BD \right] \approx\sigma_{d}\mathbb{E}\left[ \omega_{B}B^{2} \right]$$

$$\approx\sigma_{d}\omega_{B}$$

$$\mathbb{E}\left[ GD \right] \approx\sigma_{d}\mathbb{E}\left[ \omega_{B}\left( \beta G^{2}+\varepsilon_{B}G \right) \right]$$

$$\approx\sigma_{d}\omega_{B}\beta$$

$$\mathbb{E}\left[ G^{2}D \right]\approx\sigma_{d}\mathbb{E}\left[ \omega_{B}\left( \beta G^{3}+\varepsilon_{B}G^{2} \right) \right]$$

$$\approx\sigma_{d}\omega_{B}\beta\mathbb{E}\left[ G^{3} \right]$$

$$\sigma_{G\left| d=1 \right.}^{2} \approx1+\sigma_{d}^{2}\omega_{B}\beta\mathbb{E}\left[ G^{3} \right]\frac{1}{\mu_{d}}-\left( \omega_{B}\beta\frac{\sigma_{d}}{\mu_{d}} \right)^{2}$$

$$\approx1+\frac{\omega_{B}\beta\sigma_{d}^{2}}{\mu_{d}}\mathbb{E}\left[ G^{3} \right]-\frac{\omega_{B}^{2}\sigma_{d}^{4}\beta^{2}}{\mu_{d}^{2}}$$

It follows that the bias can be expressed as:

$$\Delta_{a} \approx\frac{\mathbb{E}\left[ BGD \right]\frac{\sigma_{d}}{\mu_{d}}-\mathbb{E}\left[ GD \right]\mathbb{E}\left[ BD \right]\left( \frac{\sigma_{d}}{\mu_{d}} \right)^{2}}{\sigma_{G\left| d=1 \right.}^{2}}+\mathbb{E}\left[ BG \right]\frac{1-\sigma_{G\left| d=1 \right.}^{2}}{\sigma_{G\left| d=1 \right.}^{2}}$$

$$\approx\frac{\frac{\omega_{B}\beta^{2}\sigma_{d}^{2}}{\mu_{d}}\mathbb{E}\left[ G^{3} \right]-\frac{\omega_{B}^{2}\sigma_{d}^{4}\beta}{\mu_{d}^{2}}}{\sigma_{G\left| d=1 \right.}^{2}}-\frac{\frac{\omega_{B}\beta^{2}\sigma_{d}^{2}}{\mu_{d}}\mathbb{E}\left[ G^{3} \right]-\frac{\omega_{B}^{2}\sigma_{d}^{4}\beta^{3}}{\mu_{d}^{2}}}{\sigma_{G\left| d=1 \right.}^{2}}$$

$$\approx-\beta\frac{\omega_{B}^{2}\sigma_{d}^{4}\left( 1-\beta^{2} \right)}{\mu_{d}^{2}\sigma_{G\left| d=1 \right.}^{2}}$$

$$\approx-\beta\frac{\omega_{B}^{2}\left( 1-\beta^{2} \right)\left( 1-\mu_{d} \right)^{2}}{\sigma_{G\left| d=1 \right.}^{2}}$$

For model *b)*, $\omega_{B}=\gamma=0$ and $\mathbb{E}\left[ BG \right]=\beta$:

$$\mathbb{E}\left[ BGD \right] \approx\sigma_{d}\mathbb{E}\left[ BG \left( \omega_{G}G \right) \right]$$

$$\approx\omega_{G}\sigma_{d}\mathbb{E}\left[ BG^{2} \right]$$

$$\approx\omega_{G}\sigma_{d}\mathbb{E}\left[ \left( \beta G+\varepsilon_{B} \right)G^{2} \right]$$

$$\approx\omega_{G}\sigma_{d}\beta\mathbb{E}\left[ G^{3} \right]$$

$$\mathbb{E}\left[ BD \right] \approx\sigma_{d}\omega_{G}\beta$$

$$\mathbb{E}\left[ GD \right] \approx\sigma_{d}\omega_{G}$$

$$\sigma_{G\left| d=1 \right.}^{2} \approx1+\omega_{G}\mathbb{E}\left[ G^{3} \right]\frac{\sigma_{d}^{2}}{\mu_{d}}-\left( \omega_{G}\frac{\sigma_{d}^{2}}{\mu_{d}} \right)^{2}$$

$$\Delta_{b} \approx\frac{\mathbb{E}\left[ BGD \right]\frac{\sigma_{d}}{\mu_{d}}-\mathbb{E}\left[ GD \right]\mathbb{E}\left[ BD \right]\left( \frac{\sigma_{d}}{\mu_{d}} \right)^{2}}{\sigma_{G\left| d=1 \right.}^{2}}+\mathbb{E}\left[ BG \right]\frac{1-\sigma_{G\left| d=1 \right.}^{2}}{\sigma_{G\left| d=1 \right.}^{2}}$$

$$\approx\frac{\frac{\omega_{G}\beta\sigma_{d}^{2}}{\mu_{d}}\mathbb{E}\left[ G^{3} \right]-\frac{\omega_{B}^{2}\sigma_{d}^{4}\beta}{\mu_{d}^{2}}-\frac{\omega_{G}\beta\sigma_{d}^{2}}{\mu_{d}}\mathbb{E}\left[ G^{3} \right]+\frac{\omega_{B}^{2}\sigma_{d}^{4}\beta}{\mu_{d}^{2}}}{\sigma_{G\left| d=1 \right.}^{2}}$$

$$\approx0$$

For model *c)* $\beta=\gamma=0$:

The independence between $B$ and $G$ implies $\mathbb{E}\left[ GB \right]=\mathbb{E}\left[ GB^{2} \right]=\mathbb{E}\left[ G^{2}B \right]=0$. We have therefore the following approximation:

$$\mathbb{E}\left[ BD \right] \approx\sigma_{d}\omega_{G}\mathbb{E}\left[ B^{2} \right]$$

$$\approx\sigma_{d}\omega_{B}$$

$$\mathbb{E}\left[ GD \right] \approx\sigma_{d}\omega_{G}\mathbb{E}\left[ G^{2} \right]$$

$$\approx\sigma_{d}\omega_{G}$$

$$\mathbb{E}\left[ G^{2}D \right] \approx\sigma_{d}\omega_{G}\mathbb{E}\left[ G^{3} \right]$$

$$\approx\sigma_{d}\omega_{G}\frac{1-\mu_{g}}{\sigma_{g}}$$

$$\sigma_{G\left| d=1 \right.}^{2} \approx1+\mathbb{E}\left[ G^{2}D \right]\frac{\sigma_{d}}{\mu_{d}}-\left( \mathbb{E}\left[ GD \right]\frac{\sigma_{d}}{\mu_{d}} \right)^{2}$$

$$\approx1+\frac{\omega_{G}\left( 1-\mu_{g} \right)}{\sigma_{g}\mu_{d}}-\left( \frac{\omega_{G}}{\mu_{d}} \right)^{2}$$

$$\Delta_{c} \approx\frac{\frac{\sigma_{d}^{2}}{\mu_{d}}\left( \omega_{B}\mathbb{E}\left[ GB^{2} \right]+\omega_{G}\mathbb{E}\left[ G^{2}B \right] \right)-\mathbb{E}\left[ GD \right]\mathbb{E}\left[ BD \right]\left( \frac{\sigma_{d}}{\mu_{d}} \right)^{2}}{\sigma_{G\left| d=1 \right.}^{2}}+\mathbb{E}\left[ BG \right]\frac{1-\sigma_{G\left| d=1 \right.}^{2}}{\sigma_{G\left| d=1 \right.}^{2}}$$

$$\approx-\frac{\mathbb{E}\left[ GD \right]\mathbb{E}\left[ BD \right]\left( \frac{\sigma_{d}}{\mu_{d}} \right)^{2}}{\sigma_{G\left| d=1 \right.}^{2}}$$

$$\approx-\omega_{G}\omega_{B}\frac{\sigma_{d}^{4}}{\mu_{d}^{2}\sigma_{G\left| d=1 \right.}^{2}}$$

$$\approx-\omega_{G}\omega_{B}\frac{\left( 1-\mu_{d} \right)^{2}}{\sigma_{G\left| d=1 \right.}^{2}}$$

For model *d)* $\omega_{B}=\beta=0$ .

This is the simplest scenario and a case well established in the literature. $D$ is a direct risk factor of $B$ and its effect is invariant with $g$. This relationship can be expressed through the linear model:

$$\mathbb{E}\left[ B|D,G \right]=\gamma D+\varepsilon_{B}$$

It follows that:

$$\mathbb{E}\left[ B|D=1,G \right]=\gamma$$

indicating that $B$ is invariant with $g$ in case only sample.

$$\Delta_{b} =-\mathbb{E}\left[ BG \right]$$

$$\approx-\gamma\mathbb{E}\left[ DG \right]$$

$$\approx-\gamma\omega_{G}\sigma_{d}$$

In our case, almost all assumptions are violated, so that an accurate estimation of the bias is not possible with the above approximation as the magnitude of $\Delta$ will in general be misspecified. Nevertheless, as aforementioned, the signs of the estimates in the linear approximation of the conditional mean of $D$ remain valid. It follows that the direction of the bias can be inferred from the above estimators independently of the distribution of $B$ and $G$.To confirm the validity of this approximation, we performed series of simulations across a broad range of scenarios, assuming rare or common disease, moderate or large effects, and drawing the residual of $B$ from either a normal distribution, an exponential distribution, or a uniform distribution. Our inference on the direction of the bias was validated in all scenarios considered (**S9-12 Figs**).

# Without the linear approximation of the Logistic model

The formal derivation of the bias using the logistic formulation that we explored led to intractable solutions. For example, re-writing the exponential function in the logistic model as power series we have:

$$\mathbb{E}\left[ d|B,G \right]\approx\frac{e^{\omega_{0}+\omega_{B}B+\omega_{G}G}}{1+e^{\omega_{0}+\omega_{B}B+\omega_{G}G}}=\frac{p_{0}\sum_{k=0}^{\infty} \frac{\left( \omega_{B}B \right)^{k}}{k!}\sum_{k=0}^{\infty} \frac{\left( \omega_{G}G \right)^{k}}{k!}}{1+p_{0}\sum_{k=0}^{\infty} \frac{\left( \omega_{B}B \right)^{k}}{k!}\sum_{k=0}^{\infty} \frac{\left( \omega_{G}G \right)^{k}}{k!}}$$

The analytical form of $\mathbb{E}\left[ GBD \right]$, $\mathbb{E}\left[ GD \right]$, and $\mathbb{E}\left[ BD \right]$ based on the above conditional expected value of $d$, implies solving the expectation of a ratio of functions including moments of $G$ and $B$ from 0 to infinity.

Instead we explored solutions leveraging the rare disease assumption to approximate the logit function with an exponential, while using the same power decomposition. However, this solution was unsuccessful, leading in particular to an expected null bias for model c) and d), in disagreement with all simulations we conducted (see e.g. **S9-12 Figs**), and was therefore not pursued further. Indeed, following this path for model c) and d) we have:

$$\mathbb{E}\left[ d|B,G \right]\approx e^{\omega_{0}+\omega_{B}B+\omega_{G}G}\approx p_{0}\sum_{k=0}^{\infty} \frac{\omega_{B}^{k}B^{k}}{k!}\sum_{k=0}^{\infty} \frac{\omega_{G}^{k}G^{k}}{k!}$$

so that,

$$\mathbb{E}\left[ D|G,B \right]\mathbb{=E}\left[ \frac{d-\mu_{d}}{\sigma_{d}}|B,G \right]$$

$$\approx\frac{p_{0}}{\sigma_{d}}\sum_{k=0}^{\infty} \frac{\omega_{B}^{k}B^{k}}{k!}\sum_{k=0}^{\infty} \frac{\omega_{G}^{k}G^{k}}{k!}-\frac{\mu_{d}}{\sigma_{d}}$$

$$var\left( d \right)=\mathbb{E}\left[ d^{2} \right]-{\mathbb{E}\left[ d \right]}^{2}$$

$$=p_{0}\sum_{k=0}^{\infty} \frac{\omega_{B}^{k}\mathbb{E}\left[ B^{k} \right]}{k!} \sum_{k=0}^{\infty} \frac{\omega_{G}^{l}\mathbb{E}\left[ G^{l} \right]}{k!}-\mu_{d}^{2}$$

$$\approx p_{0}\sum_{k=0}^{\infty} \frac{\omega_{B}^{k}\mathbb{E}\left[ B^{k} \right]}{k!} \sum_{k=0}^{\infty} \frac{\omega_{G}^{l}\mathbb{E}\left[ G^{l} \right]}{k!}$$

$$\mathbb{E}\left[ BGD \right]=\mathbb{E}\left[ BG\left( \frac{p_{0}}{\sigma_{d}}\sum_{k=0}^{\infty} \frac{\left( \omega_{B}B \right)^{k}}{k!}\sum_{k=0}^{\infty} \frac{\left( \omega_{G}G \right)^{k}}{k!}-\frac{\mu_{d}}{\sigma_{d}} \right) \right]$$

$$=\frac{p_{0}}{\sigma_{d}}\sum_{k=0}^{\infty} \frac{\omega_{B}^{k}\mathbb{E}\left[ B^{k+1} \right]}{k!}\sum_{k=0}^{\infty} \frac{\omega_{G}^{l}\mathbb{E}\left[ G^{l+1} \right]}{k!}$$

$$\mathbb{E}\left[ GD \right] =\frac{p_{0}}{\sigma_{d}}\sum_{k=0}^{\infty} \frac{\omega_{B}^{k}\mathbb{E}\left[ B^{k} \right]}{k!}\sum_{k=0}^{\infty} \frac{\omega_{G}^{l}\mathbb{E}\left[ G^{l+1} \right]}{k!}$$

$$\mathbb{E}\left[ BD \right] =\frac{p_{0}}{\sigma_{d}}\sum_{k=0}^{\infty} \frac{\omega_{B}^{k}\mathbb{E}\left[ B^{k+1} \right]}{k!}\sum_{k=0}^{\infty} \frac{\omega_{G}^{l}\mathbb{E}\left[ G^{l} \right]}{k!}$$

$$\Delta=\frac{\mathbb{E}\left[ BGD \right]\frac{\sigma_{d}}{\mu_{d}}-\mathbb{E}\left[ GD \right]\mathbb{E}\left[ BD \right]\left( \frac{\sigma_{d}}{\mu_{d}} \right)^{2}}{\sigma_{g\left| d=1 \right.}}$$

$$=\frac{\mathbb{E}\left[ BGD \right]\frac{\sigma_{d}}{\mu_{d}}-\mathbb{E}\left[ GD \right]\mathbb{E}\left[ BD \right]\left( \frac{\sigma_{d}}{\mu_{d}} \right)^{2}}{\sigma_{g\left| d=1 \right.}}$$

$$=\left( \frac{p_{0}}{\mu_{d}}\sum_{k=0}^{\infty} \frac{\omega_{B}^{k}\mathbb{E}\left[ B^{k+1} \right]}{k!}\sum_{k=0}^{\infty} \frac{\omega_{G}^{l}\mathbb{E}\left[ G^{l+1} \right]}{k!}-\frac{p_{0}^{2}}{\mu_{d}^{2}}\sum_{k=0}^{\infty} \frac{\omega_{B}^{k}\mathbb{E}\left[ B^{k+1} \right]}{k!}\sum_{k=0}^{\infty} \frac{\omega_{G}^{l}\mathbb{E}\left[ G^{l+1} \right]}{k!}\sum_{k=0}^{\infty} \frac{\omega_{B}^{k}\mathbb{E}\left[ B^{k} \right]}{k!}\sum_{k=0}^{\infty} \frac{\omega_{G}^{l}\mathbb{E}\left[ G^{l} \right]}{k!} \right) \frac{1}{\sigma_{g\left| d=1 \right.}}$$

$$=\frac{p_{0}}{\mu_{d}}\sum_{k=0}^{\infty} \frac{\omega_{B}^{k}\mathbb{E}\left[ B^{k+1} \right]}{k!}\sum_{k=0}^{\infty} \frac{\omega_{G}^{l}\mathbb{E}\left[ G^{l+1} \right]}{k!}\left( 1-\frac{p_{0}}{\mu_{d}}\sum_{k=0}^{\infty} \frac{\omega_{B}^{k}\mathbb{E}\left[ B^{k} \right]}{k!}\sum_{k=0}^{\infty} \frac{\omega_{G}^{l}\mathbb{E}\left[ G^{l} \right]}{k!} \right) \frac{1}{\sigma_{g\left| d=1 \right.}}$$

$$=\frac{p_{0}}{\mu_{d}}\sum_{k=0}^{\infty} \frac{\omega_{B}^{k}\mathbb{E}\left[ B^{k+1} \right]}{k!}\sum_{k=0}^{\infty} \frac{\omega_{G}^{l}\mathbb{E}\left[ G^{l+1} \right]}{k!}\left( 1-\frac{\mu_{d}}{\mu_{d}} \right) \frac{1}{\sigma_{g\left| d=1 \right.}}$$

$$=0$$

# Alternative study design

Using these IBD-cases samples to explore causal relationship has some advantages over other study designs. Study on healthy subjects can only be used to test for association between genetic variants and bacteria but cannot be used to decipher potential mediation effect. On the other hand, while prospective cohorts in the general population have the potential to solve a number of statistical artifacts faced in our study, they require unrealistic sample size when studying relatively rare disease like IBD. A more reasonable alternative to IBD-cases only is case-control data. Case-control data provide additional information on SNP-bacteria association while only requiring a reasonable increase the sample size (e.g. matched healthy subjects). Nevertheless, case-control data does not solve the selection bias issue faced in the present study. By definition there is no simple and general solution to address selection bias. Instead the evaluation of its impact has to be done on a case by case basis after accounting for the correlation between variables in the general population, as done in our study. Our extensive simulations and sensitivity analyses confirm the robustness of our inference for the range of effect size considered.
