# Supplementary material for: Genetic effects on the commensal microbiota in inflammatory bowel disease patients
Source: PLoS Genet. 2019 Mar 8;15(3):e1008018. doi: 10.1371/journal.pgen.1008018 (PMC6426259; doi:10.1371/journal.pgen.1008018)
Supplement: S1 Table — (DOCX) [file pgen.1008018.s002.docx]

# S1 Table. Characteristics of the genetic variants analyzed

| **Gene** | **RSid** | **A_0_/A_1_** | **RAF** | **Genotype count** | | | **N** |
| --- | --- | --- | --- | --- | --- | --- | --- |
|  |  |  |  | **A_0_A_0_** | **A_0_A_1_** | **A_1_A_1_** |  |
| **ATG16L1** | rs12994997 | G/A | 0.542 | 39 | 85 | 54 | 178 |
| **CARD9** | rs10781499 | G/A | 0.438 | 53 | 93 | 31 | 177 |
| **LRRK2** | rs11564258 | G/A | 0.051 | 159 | 18 | 0 | 177 |
| **NOD2 (R702W)** | rs2066844 | C/T | 0.054 | 159 | 17 | 1 | 177 |
| **NOD2 (G908R)** | rs2066845 | G/C | 0.042 | 164 | 15 | 0 | 179 |
| **NOD2 (1007FS)** | rs2066847 | -/C | 0.022 | 175 | 4 | 2 | 181 |

**A_0_/A_1_: protective allele/risk allele for IBD; RAF: IBD risk allele frequency. The IL23R variant was not included in the final analysis because of low allele frequency.*
