# Supplementary material for: Genetic effects on the commensal microbiota in inflammatory bowel disease patients
Source: PLoS Genet. 2019 Mar 8;15(3):e1008018. doi: 10.1371/journal.pgen.1008018 (PMC6426259; doi:10.1371/journal.pgen.1008018)
Supplement: S2 Table — (DOCX) [file pgen.1008018.s003.docx]

# S2 Table. Significant association within each hierarchical level

| **Level** | **Outcome** | **Gene** | **N** | ***(carrier)^a^*** | ***β^b^*** | ***(SE)*** | **P-value** |
| --- | --- | --- | --- | --- | --- | --- | --- |
| Phylum | *Firmicutes* | *CARD9* | 182 | *(155)* | -0.38 | *(0.10)* | 2.3x10^-4^ |
| Class | *Bacteroidia* | *NOD2* | 182 | *(36)* | -0.54 | *(0.16)* | 8.9x10^-4^ |
| Family | *Bacteroidaceae* | *NOD2* | 182 | *(36)* | -0.62 | *(0.16)* | 1.0x10^-4^ |
| Genus | *Bacteroides* | *NOD2* | 182 | *(36)* | -0.62 | *(0.16)* | 1.9x10^-4^ |
|  | *Roseburia* | *NOD2* | 182 | *(36)* | -0.58 | *(0.14)* | 6.4x10^-5^ |
| Species | *Faecis* | *NOD2* | 182 | *(36)* | -0.59 | *(0.16)* | 2.3x10^-4^ |
|  | *Prausnitzii* | *NOD2* | 182 | *(36)* | -0.56 | *(0.16)* | 4.0x10^-4^ |

***^a^****N is the count of individuals per sub-group, while carrier corresponds to the number of individuasl carrying a least one of the risk allele within that sub-group*

*^b^Beta coefficient are derived while using the allele associated with an increased risk of IBD as the coded allele. Outcomes were normalized and all have a variance of 1, while genetic variants were not transformed, so that beta coefficient correspond to the estimated change in the outcome mean per additional risk allele.*
