# Supplementary material for: Genetic effects on the commensal microbiota in inflammatory bowel disease patients
Source: PLoS Genet. 2019 Mar 8;15(3):e1008018. doi: 10.1371/journal.pgen.1008018 (PMC6426259; doi:10.1371/journal.pgen.1008018)
Supplement: S3 Table — (DOCX) [file pgen.1008018.s004.docx]

# S3 Table. Type I error rate under four hypothetical models

| **Model** | **Disease Prevalence** | **True** $\boldsymbol{\beta}_{\boldsymbol{G}}$ | ${\hat{\boldsymbol{\beta}}}_{\boldsymbol{G}}$ | $\boldsymbol{\sigma}_{{\hat{\boldsymbol{\beta}}}_{\boldsymbol{G}}}$ | $\tilde{\boldsymbol{pval}}$ | $\frac{\sum\boldsymbol{pval<0.05}}{\boldsymbol{N}_{\boldsymbol{rep}}}$ |
| --- | --- | --- | --- | --- | --- | --- |
| ***a)*** | 0.010 | -0.316 | -0.307 | 0.130 | 0.019 | 0.654 |
| ***b)*** | 0.010 | 0 | 0.005 | 0.089 | 0.500 | 0.050 |
| ***c)*** | 0.010 | 0 | 0.034 | 0.119 | 0.479 | 0.050 |
| ***d)*** | 0.010 | 0 | 0.038 | 0.120 | 0.451 | 0.068 |

*Disease Prevalence is the mean of the prevalence across all replicates ;* $\hat{\beta}_{G}$ *is the mean of the estimated effect of the genetic variant on the bacterium, and* $\sigma_{{\hat{\boldsymbol{\beta}}}_{\boldsymbol{G}}}$ *its variance across all replicates.* $\tilde{pval}$ *is the median of the p-value across all replicates.* $N_{rep}$*, the number of replicate, equals 10,000.*
