# Supplementary material for: Genetic effects on the commensal microbiota in inflammatory bowel disease patients
Source: PLoS Genet. 2019 Mar 8;15(3):e1008018. doi: 10.1371/journal.pgen.1008018 (PMC6426259; doi:10.1371/journal.pgen.1008018)
Supplement: S4 Table — (DOCX) [file pgen.1008018.s005.docx]

# S4 Table. Characteristics of the replication studies

|  |  | **Replication 1 (MGH)** | | | |  | **Replication 2 (MSH)** | | | |  | **Replication 3 (Neth)** | | | |
| --- | --- | --- | --- | --- | --- | --- | --- | --- | --- | --- | --- | --- | --- | --- | --- |
|  |  | ***N (%)*** | | ***Missing (%)*** | |  | ***N (%)*** | | ***Missing (%)*** | |  | ***N (%)*** | | ***Missing (%)*** | |
| **N total** |  | 170 |  |  |  |  | 65 |  |  |  |  | 216 |  |  |  |
| **Mean age** |  | 40.2 |  | 0 | *(0%)* |  | 33.9 |  | *0* | *(0%)* |  | 43.5 |  | 0 | *(0%)* |
| **Female** |  | 94 | *(55%)* | 0 | *(0%)* |  | 34 | *(52%)* | *0* | *(0%)* |  | 137 | *(63%)* | 0 | *(0%)* |
| **Mesalamine** |  | 47 | *(28%)* | 33 | *(19%)* |  | 34 | *(52%)* | *3* | *(5%)* |  | 90 | *(42%)* | 10 | *(5%)* |
| **Antibiotics** |  | 5 | *(3%)* | 0 | *(0%)* |  | 7 | *(11%)* | *0* | *(0%)* |  | 0 | *(0%)* | 10 | *(5%)* |
| **Immunossupressant** |  | 107 | *(63%)* | 0 | *(0%)* |  | 3 | *(5%)* | *1* | *(2%)* |  | 90 | *(42%)* | 10 | *(5%)* |
| **Steroids** |  | 34 | *(20%)* | 33 | *(19%)* |  | 4 | *(7%)* | *7* | *(11%)* |  | 42 | *(19%)* | 10 | *(5%)* |
| **ThiopurineMtx** |  | - |  | 170 | *(100%)* |  |  | - | *65* | *(100%)* |  | 82 | *(38%)* | 0 | *(0%)* |
| **Smoker** |  |  |  | 33 | *(19%)* |  |  |  | *1* | *(2%)* |  |  |  | 3 | *(1%)* |
| ***current*** |  | 12 | *(7%)* | - |  |  | 6 | *(9%)* |  | - |  | 48 | *(22%)* |  |  |
| ***never*** |  | 79 | *(46%)* | - |  |  | 47 | *(72%)* |  | - |  | - |  | - |  |
| ***previous*** |  | 46 | *(27%)* | - |  |  | 11 | *(17%)* |  | - |  | - |  | - |  |
| ***non-smoker*** |  | 125 | *(73%)* |  |  |  |  |  |  |  |  | 165 | *(76%)* |  |  |
| **Disease** |  |  |  | 0 | *(0%)* |  |  |  | *0* | *(0%)* |  |  |  | 18 | *(8%)* |
| ***CD*** |  | 105 | *(62%)* | - |  |  | 29 | *(45%)* |  | - |  | 114 | *(53%)* | - |  |
| ***UC*** |  | 61 | *(36%)* | - |  |  | 29 | *(45%)* |  | - |  | 84 | *(39%)* | - |  |
| ***HC*** |  | 0 | *(0%)* | - |  |  | 2 | *(3%)* |  | - |  | - |  | - |  |
| ***IC*** |  | 4 | *(2%)* | - |  |  | 5 | *(8%)* |  | - |  | - |  | - |  |

*Abbreviation. MGH: Mass General Hospital; MSH: Mount Sinai Hospital ; Netherland*
