# Supplementary material for: Genetic effects on the commensal microbiota in inflammatory bowel disease patients
Source: PLoS Genet. 2019 Mar 8;15(3):e1008018. doi: 10.1371/journal.pgen.1008018 (PMC6426259; doi:10.1371/journal.pgen.1008018)
Supplement: S5 Table — (DOCX) [file pgen.1008018.s006.docx]

# S5 Table. Replication analysis per cohort

| **Outcome** | **Gene** | **Disease** | **Discovery** | | **Replication 1** | | **Replication 2** | | **Replication 3** | | **Meta** |
| --- | --- | --- | --- | --- | --- | --- | --- | --- | --- | --- | --- |
|  |  |  | ***β^*^*** | ***(Pval)*** | ***β^*^*** | ***(Pval)*** | ***β^*^*** | ***(Pval)*** | ***β^*^*** | ***(Pval)*** | ***Pval_meta_*** |
| *Firmicutes* | *CARD9* | IBD | -0.38 | *(2.3x10^-4^)* | - |  | *-* |  | -0.08 | *(0.50)* | 1.1*x10^-3^* |
|  |  | CD | -0.43 | *(7.4x10^-4^)* | - |  | *-* |  | -0.05 | *(0.76)* | 2.8*x10^-3^* |
|  |  | CDil | -0.44 | *(2.7x10^-3^)* | - |  | *-* |  | -0.08 | *(0.67)* | 6.4*x10^-3^* |
|  |  | CDni | -0.46 | *(0.23)* | - |  | *-* |  | 0.85 | *(0.080)* | 0.66 |
|  |  | UC | -0.25 | *(0.19)* | - |  | *-* |  | 0.07 | *(0.74)* | 0.46 |
| *Bacteroides* | *NOD2* | IBD | -0.62 | *(1.9x10^-4^)* | -0.28 | *(0.12)* | -0.05 | *(0.75)* | 0.21 | *(0.28)* | 0.014 |
|  |  | CD | -0.57 | *(4.1x10^-3^)* | -0.37 | *(0.083)* | -0.23 | *(0.39)* | 0.14 | *(0.57)* | 6.3*x10^-3^* |
|  |  | CDil | -0.65 | *(3.3x10^-3^)* | -0.37 | *(0.15)* | -0.17 | *(0.47)* | 0.09 | *(0.74)* | 8.7*x10^-3^* |
|  |  | CDni | - |  | -0.42 | *(0.42)* | *-* |  | - |  | 0.41 |
|  |  | UC | -0.50 | *(0.18)* | -0.01 | *(0.99)* | 0.32 | *(0.14)* | 0.15 | *(0.73)* | 0.47 |
| *Roseburia* | *NOD2* | IBD | -0.58 | *(6.4x10^-5^)* | -0.26 | *(0.16)* | -0.25 | *(0.25)* | -0.14 | *(0.48)* | 4.9*x10^-5^* |
|  |  | CD | -0.46 | *(6.7x10^-3^)* | -0.32 | *(0.19)* | -0.15 | *(0.67)* | -0.27 | *(0.25)* | 1.5*x10^-3^* |
|  |  | CDil | -0.30 | *(0.082)* | -0.38 | *(0.19)* | -0.35 | *(0.24)* | -0.30 | *(0.23)* | 5.2*x10^-3^* |
|  |  | CDni | - |  | -0.27 | *(0.62)* | *-* |  | - |  | 0.61 |
|  |  | UC | -0.76 | *(0.0052)* | 0.03 | *(0.95)* | -0.32 | *(0.40)* | 0.88 | *(0.044)* | 0.14 |
| *F. prausnitzii* | *NOD2* | IBD | -0.56 | *(4.0x10^-4^)* | -0.24 | *(0.28)* | -0.56 | *(0.072)* | -0.25 | *(0.13)* | 3.2*x10^-5^* |
|  |  | CD | -0.48 | *(6.4x10^-3^)* | -0.13 | *(0.64)* | 0.05 | *(0.92)* | -0.28 | *(0.16)* | 4.7*x10^-3^* |
|  |  | CDil | -0.51 | *(0.014)* | 0.08 | *(0.80)* | -0.04 | *(0.96)* | -0.48 | *(0.015)* | 2.0*x10^-3^* |
|  |  | CDni | - |  | -0.08 | *(0.87)* | *-* |  | - |  | 0.86 |
|  |  | UC | -0.32 | *(0.33)* | -0.12 | *(0.79)* | -0.91 | *(0.14)* | 0.24 | *(0.57)* | 0.31 |

*Abbreviation: CD, Crohn’s disease; UC, ulcerative colitis; CDil, CD ileal; CDni, CD non-ileal*
