# Supplementary figures and images for: Genetic effects on the commensal microbiota in inflammatory bowel disease patients
Source: PLoS Genet. 2019 Mar 8;15(3):e1008018. doi: 10.1371/journal.pgen.1008018 (PMC6426259; doi:10.1371/journal.pgen.1008018)

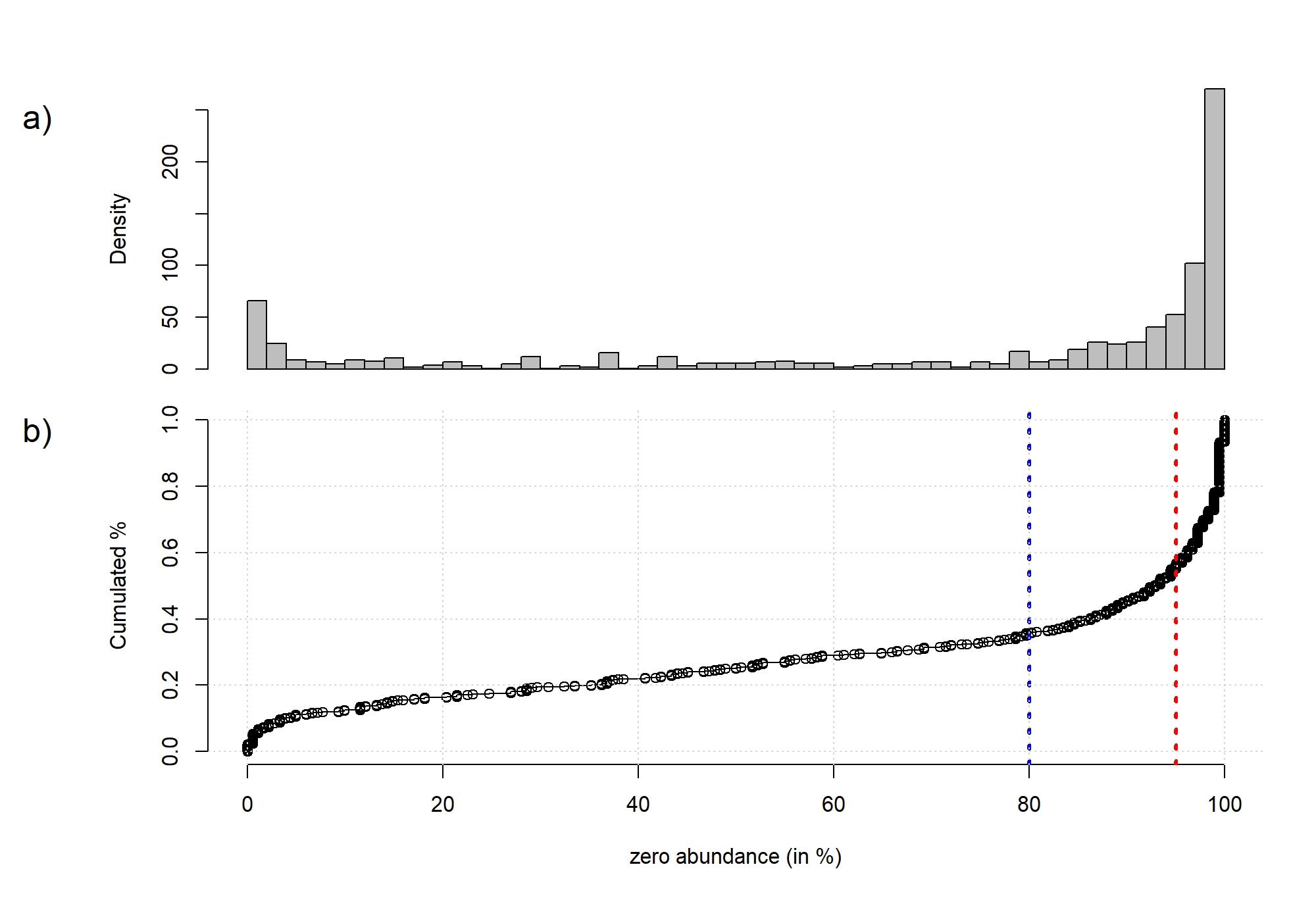

Supplement: S1 Fig — Density (a) and cumulative distribution (b) of the percentage of non-zero abundance for each of 897 bacterial taxa across the 182 IBD cases analyzed. The red and blue dashed lines in (b) correspond to a non-zero abundance of 95% and 80%, respectively. There was 494 (~55%) and 320 taxa (~36%) with lower values, respectively, i.e. taxa that are present in more than 5% and 20% of the participants, respectively. (TIF) [file pgen.1008018.s007.tif]

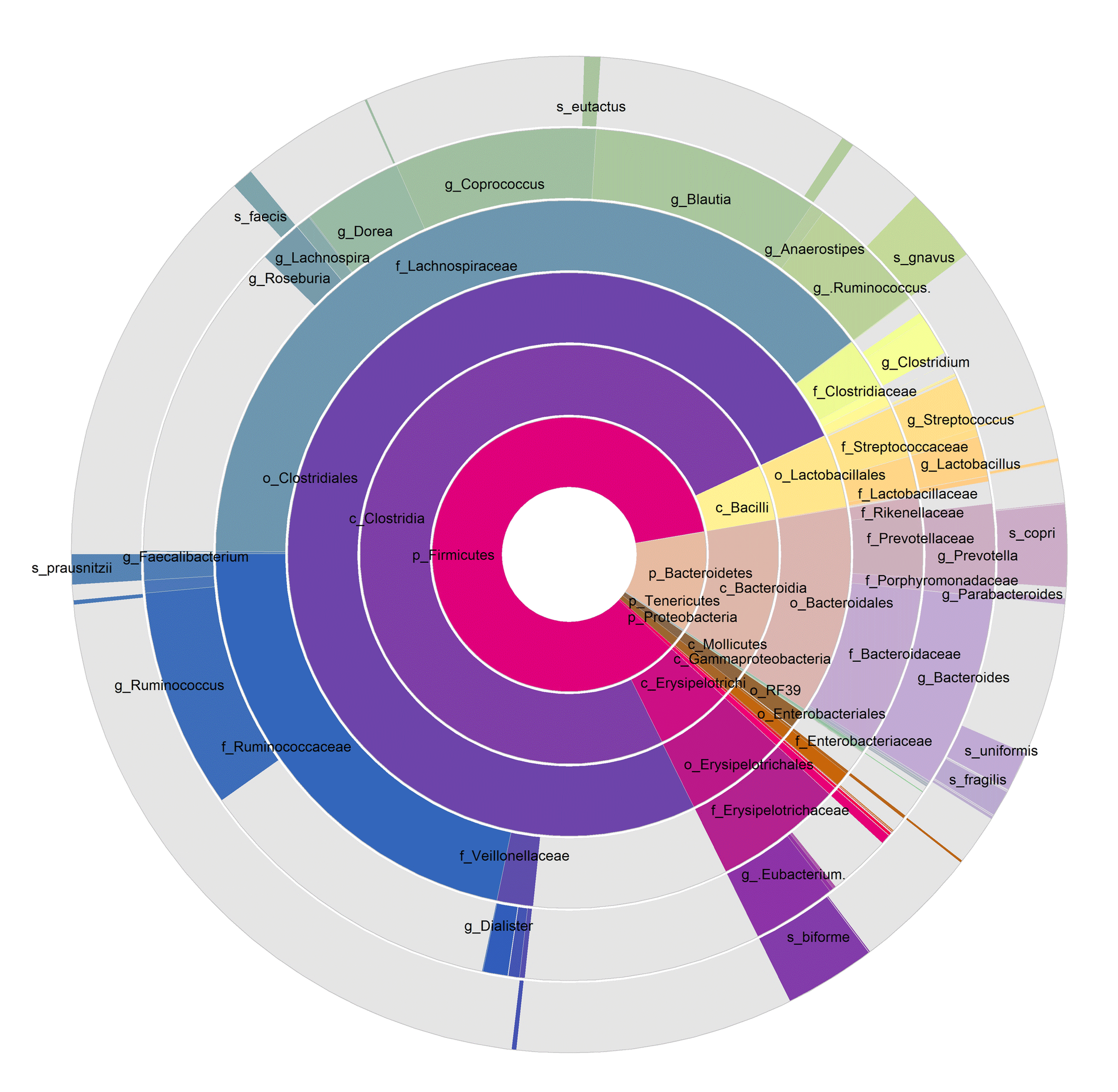

Supplement: S2 Fig — Relative proportion of each of the 168 bacterial taxa analyzed across each of the six hierarchical levels. Only taxa representing more than 0.5% of the total bacterial loading are labelled. Grey areas correspond to unknown, unmeasured, or underrepresented taxa. (TIF) [file pgen.1008018.s008.tif]

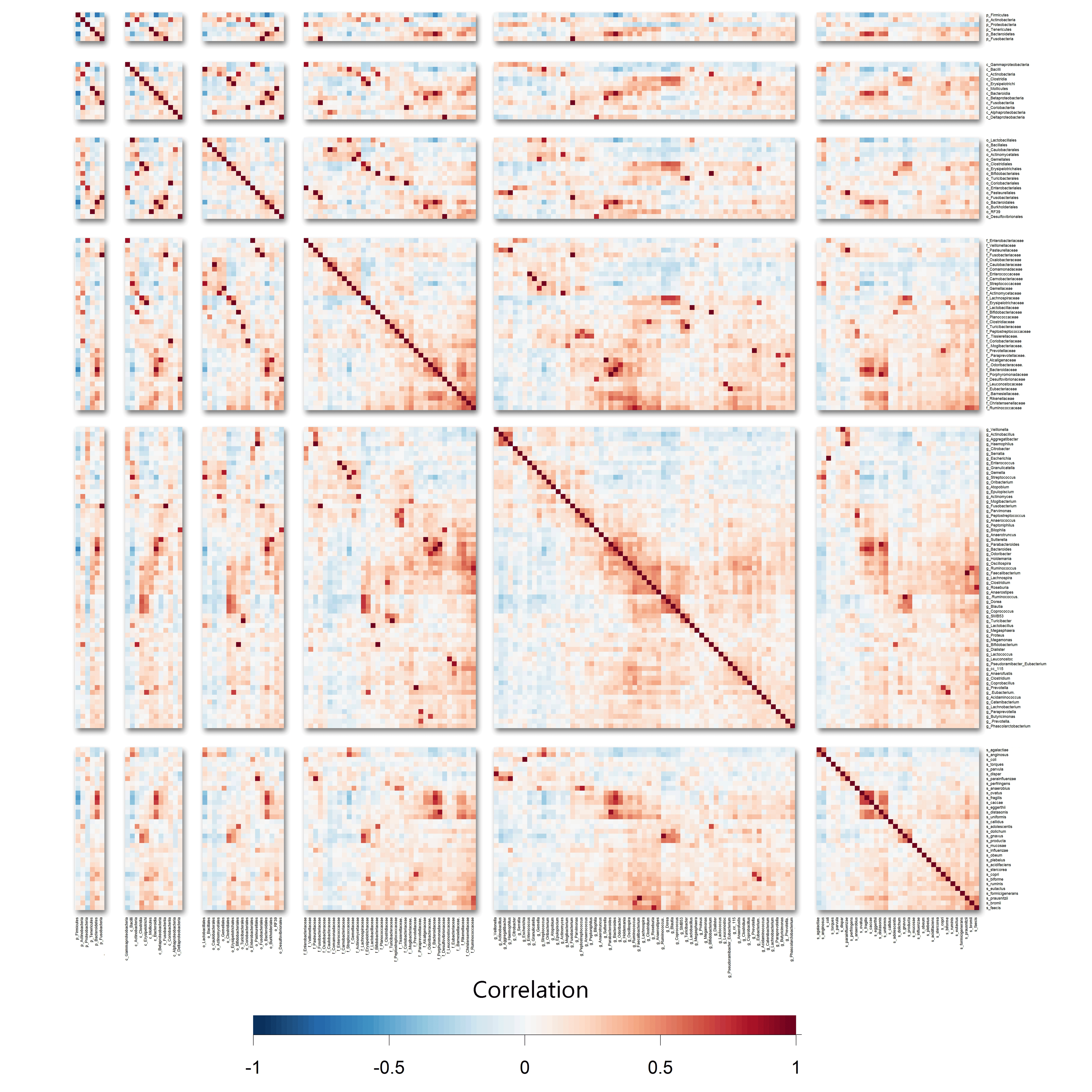

Supplement: S3 Fig — Pairwise Pearson correlation between bacterial taxa derived across the 182 IBD cases. Taxa were grouped by hierarchical strata (Phylum, Class, Order, Family, Genus and Species), so that the panels from the diagonal represent the correlation within each stratum, while off-diagonal panels present cross-strata correlation. Strength of correlation is presented as a gradient from dark blue (-1) to dark red (1). (TIF) [file pgen.1008018.s009.tif]

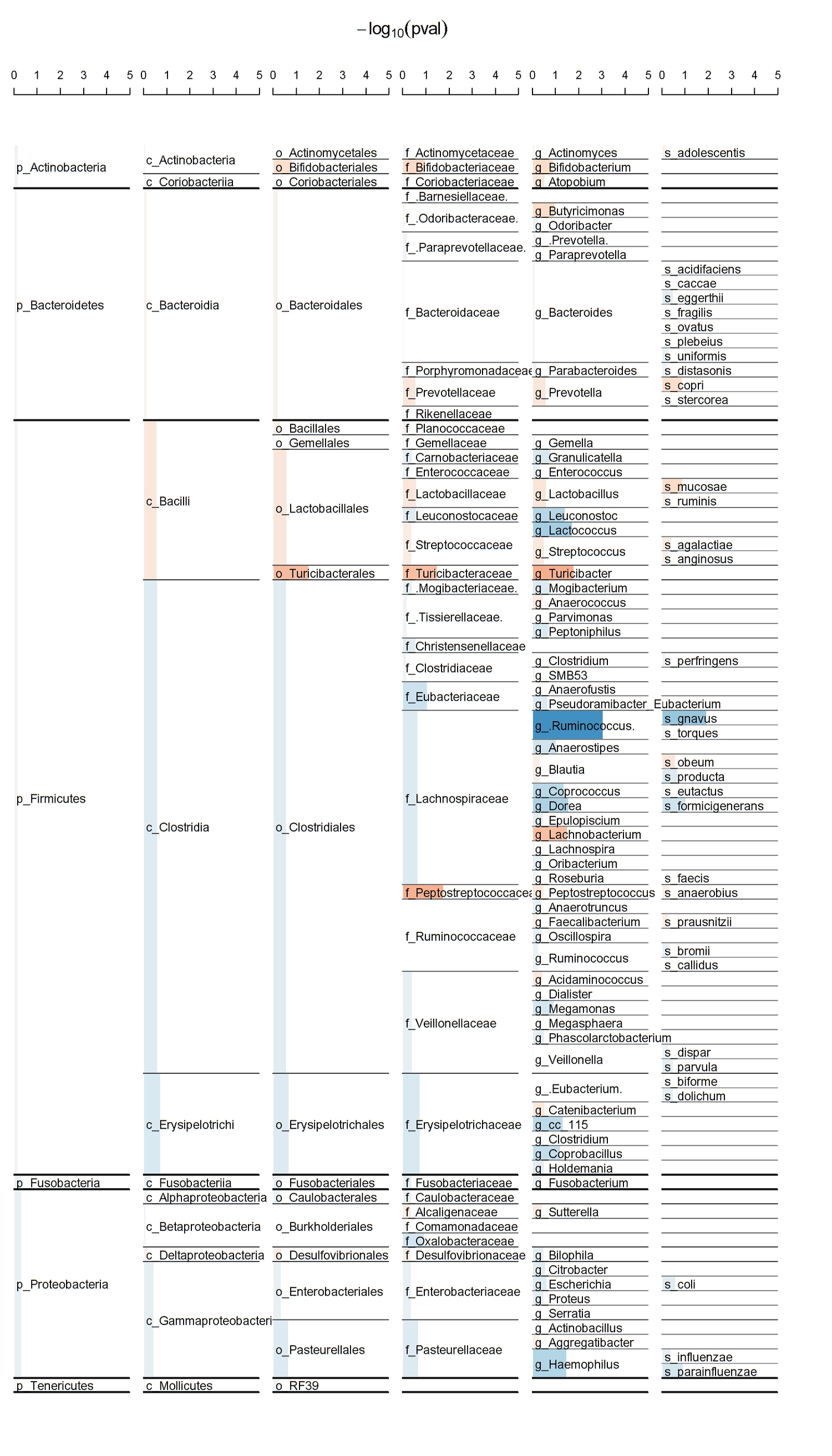

Supplement: S4 Fig — Bacterial levels were tested for association with SNP rs12994997 from gene ATG16L1. Positive and negative associations are represented as gradient of orange and blue, respectively. The horizontal axis indicates the corresponding–log10(p-value). Results are presented across the taxa hierarchy. Empty cells indicate that the subsequent element is unknown or unmeasured in our samples. (TIF) [file pgen.1008018.s010.tif]

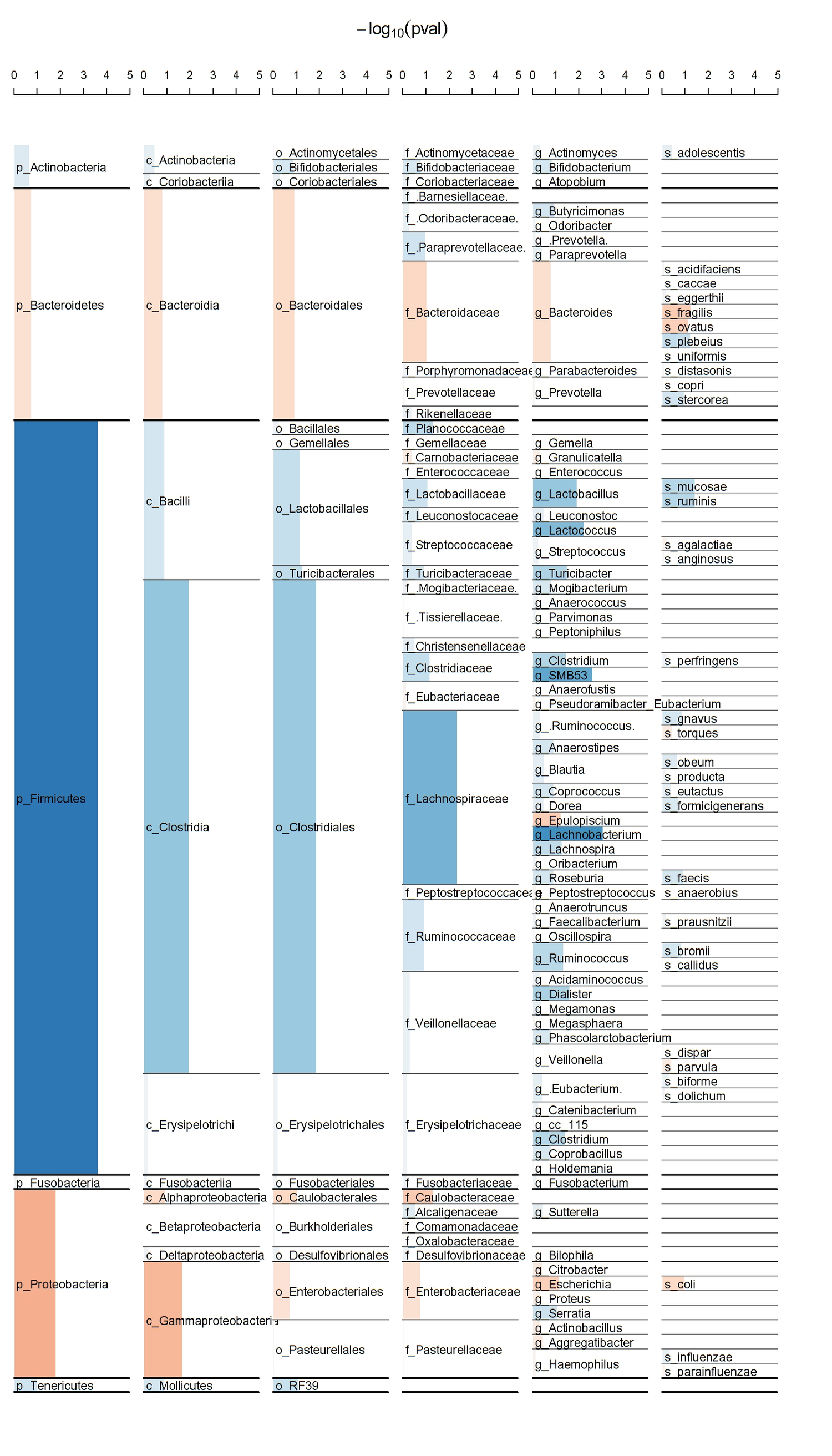

Supplement: S5 Fig — Bacterial levels were tested for association with SNP rs10781499 from gene CARD9. Positive and negative correlations are represented as gradient of orange and blue, respectively. The horizontal axis indicates the corresponding–log10(p-value). Results are presented across the taxa hierarchy. Empty cells indicate that the subsequent element is unknown or unmeasured in our samples. (TIF) [file pgen.1008018.s011.tif]

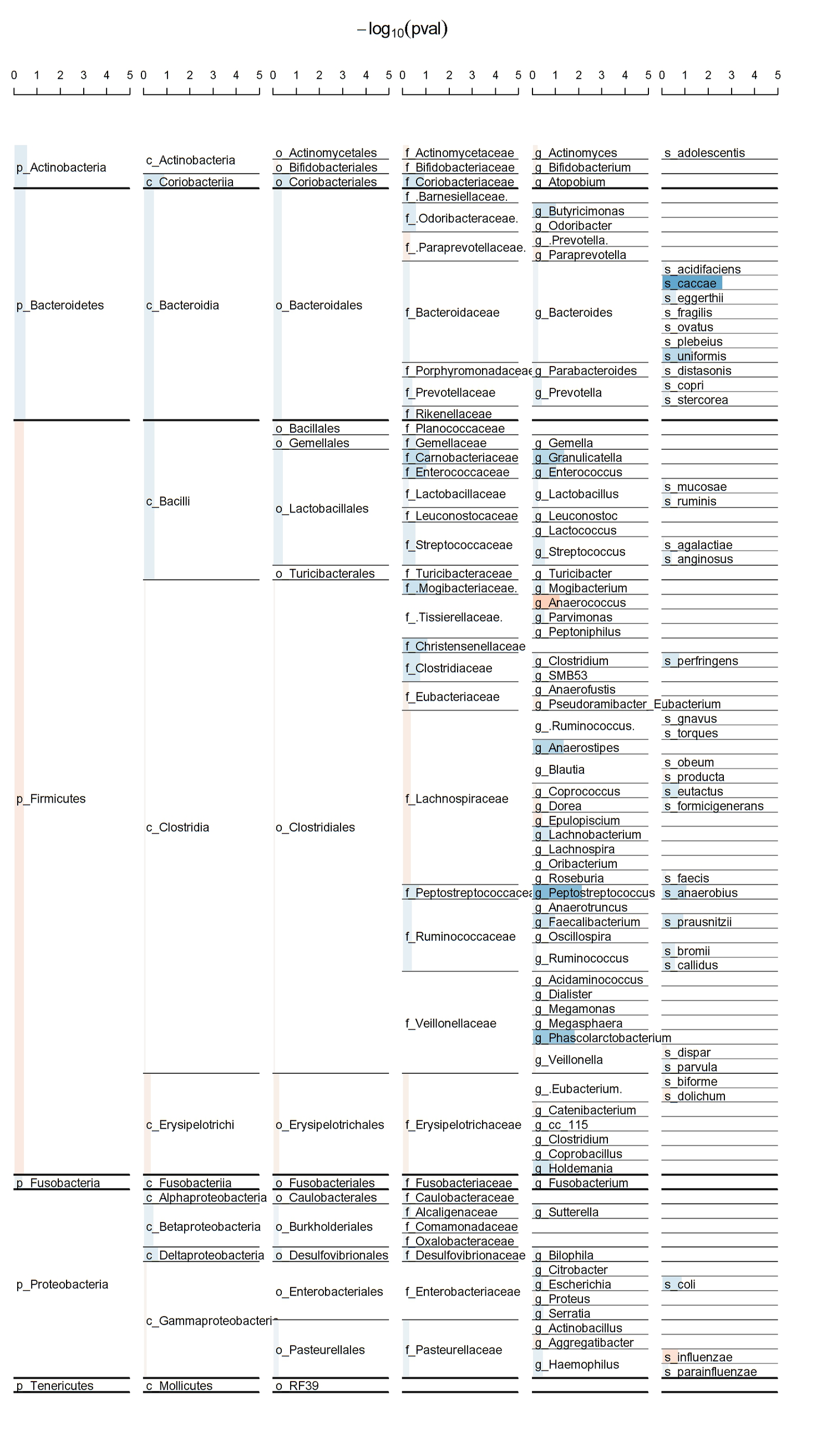

Supplement: S6 Fig — Bacterial levels were tested for association with SNP rs11564258 from gene LRRK2. Positive and negative correlations are represented as gradient of orange and blue, respectively. The horizontal axis indicates the corresponding–log10(p-value). Results are presented across the taxa hierarchy. Empty cells indicate that the subsequent element is unknown or unmeasured in our samples. (TIF) [file pgen.1008018.s012.tif]

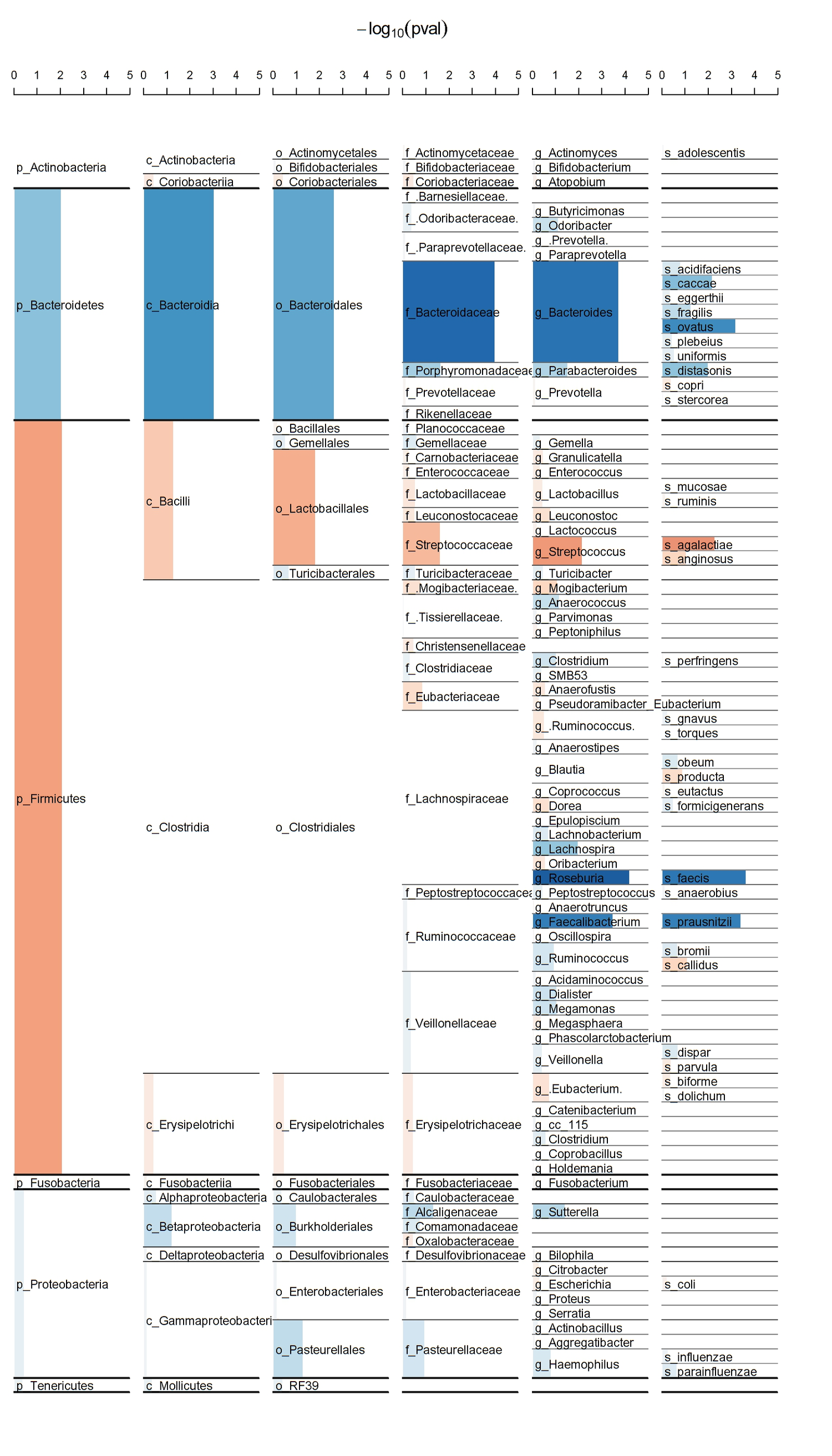

Supplement: S7 Fig — Bacterial levels were tested for association with the genetic risk score of gene NOD2 (rs2066844 + rs2066845 + rs2066847). Positive and negative correlations are represented as gradient of orange and blue, respectively. The horizontal axis indicates the corresponding–log10(p-value). Results are presented across the taxa hierarchy. Empty cells indicate that the subsequent element is unknown or unmeasured in our samples. (TIF) [file pgen.1008018.s013.tif]

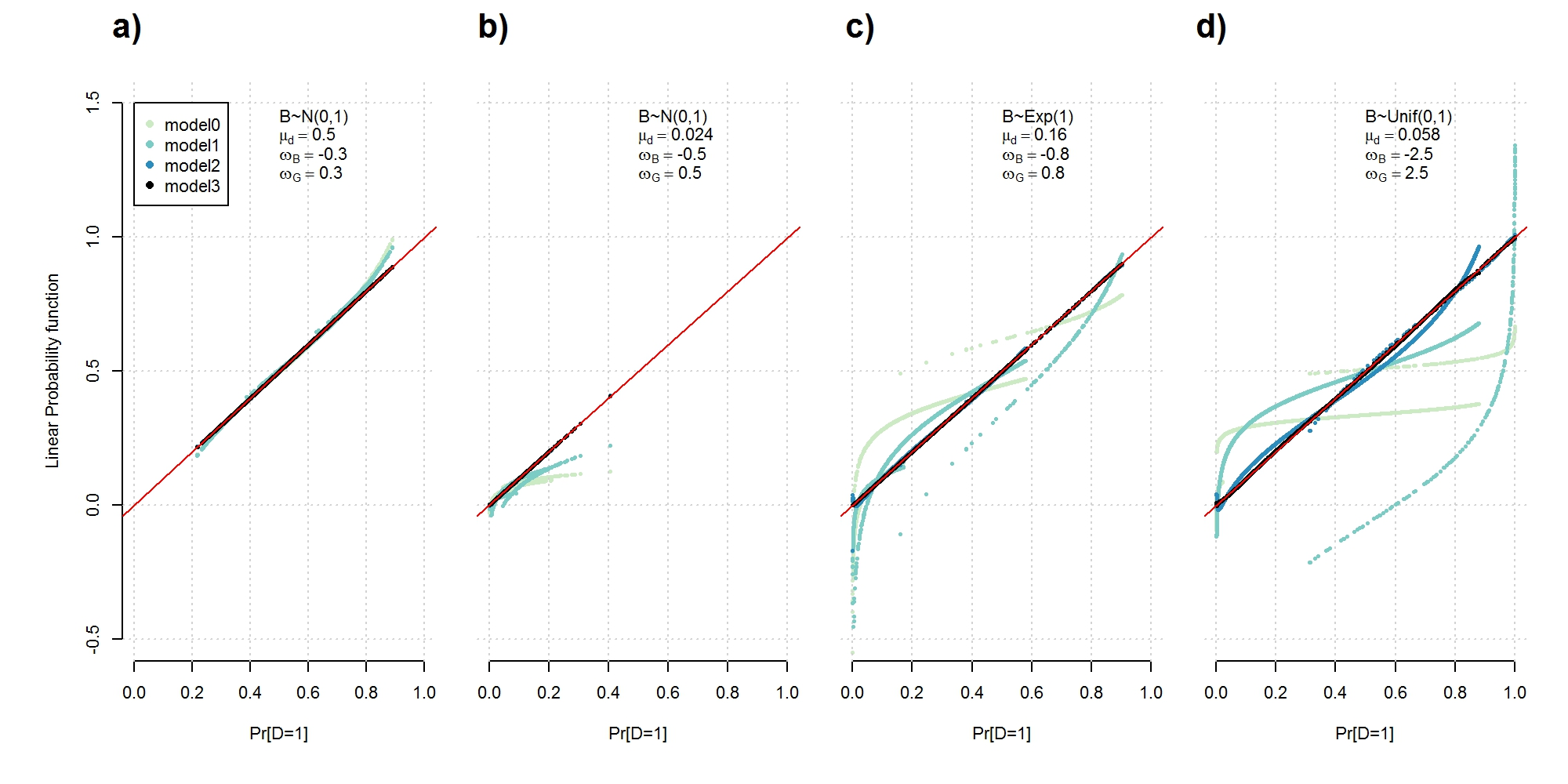

Supplement: S8 Fig — We simulated four datasets including each 20,000 individuals. For each dataset we draw a genetic variant G from a binomial assuming a minor allele frequency of 0.1, a bacterial level B while using arbitrarily various distributions, and a disease status d generated from a logistic model, E[d|B,G]=1/(1+e−[ω0+ωBB+ωGG]). We then performed linear regression of the disease probability while including an increasing number of polynomial terms of the predictors B and G and their interactions (model0 to model3). At the two extremes, model0 includes only the marginal effects of the predictor (E[d|B,G]∼λ0+λGG+λBB), while model3 include polynomial up to a power of 4 and any relevant interactions. For each model we plotted the simulated disease probability against the fitted values for model0 to model3. We use four sets of parameters to illustrate the requirement for additional terms in the linear model as the prevalence moves away from 0.5 and effect are getting larger. In (a) we considered a disease prevalence of 0.5, a normally distributed B, and modest B and G effects. In (b) we slightly increase effects and considered a rare disease case. In (c) we used generated B from an exponential, consider low disease prevalence and large effects. Finally, in (d) we considered a rare disease, very large effects and generated B from a uniform distribution. (TIF) [file pgen.1008018.s014.tif]

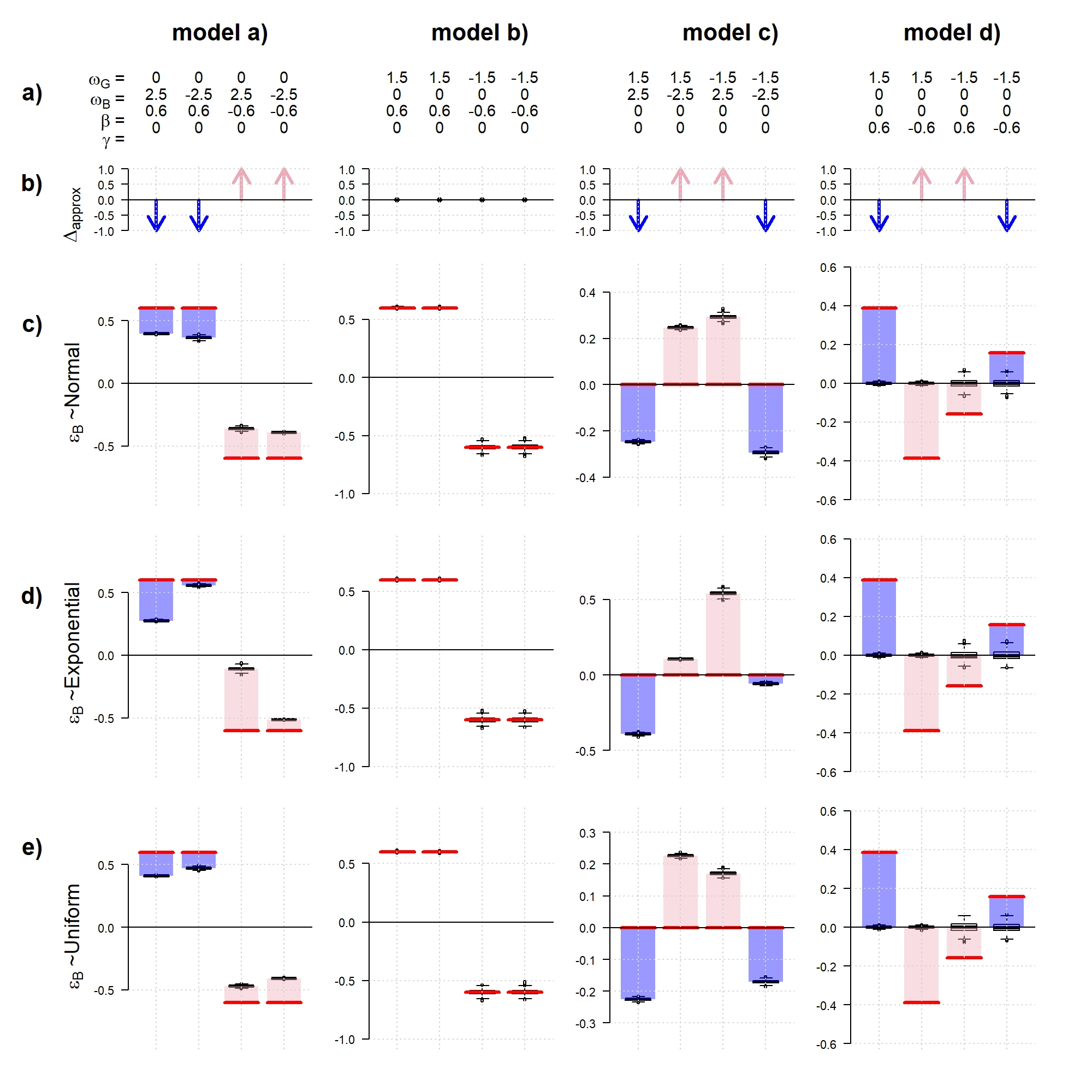

Supplement: S9 Fig — We simulated series of 1,000 replicates each including 100,000 individuals. For each replicate we draw a genetic variant G, a bacterial level B, and a disease status d using the two equations B = βG + γD + εB and E[d|B,G]=1/(1+e−[ω0+ωBB+ωGG]). We use three sets of parameters to match model a (left column), model b (middle column) and models c and d (right column), while changing the direction of the effect. In this simulation, all effects (panel a) were assumed to be large and disease prevalence high (~30%). For each simulation we estimated through standard linear regression the association coefficient between B and G in the whole population and in the cases only. Median of the coefficient in the whole population is indicated by a bold red line, while coefficients observed in cases only are provided in boxplots. The difference between the two coefficients (case only—whole population), Δ, is indicated in blue if negative and in pink if positive. For each set of parameters (panel a), we derived the proposed approximation of Δ (panel b), and the aforementioned estimates while drawing εB from a normal distribution (panel c), an exponential distribution (panel d), and a uniform distribution (panel e). (TIF) [file pgen.1008018.s015.tif]

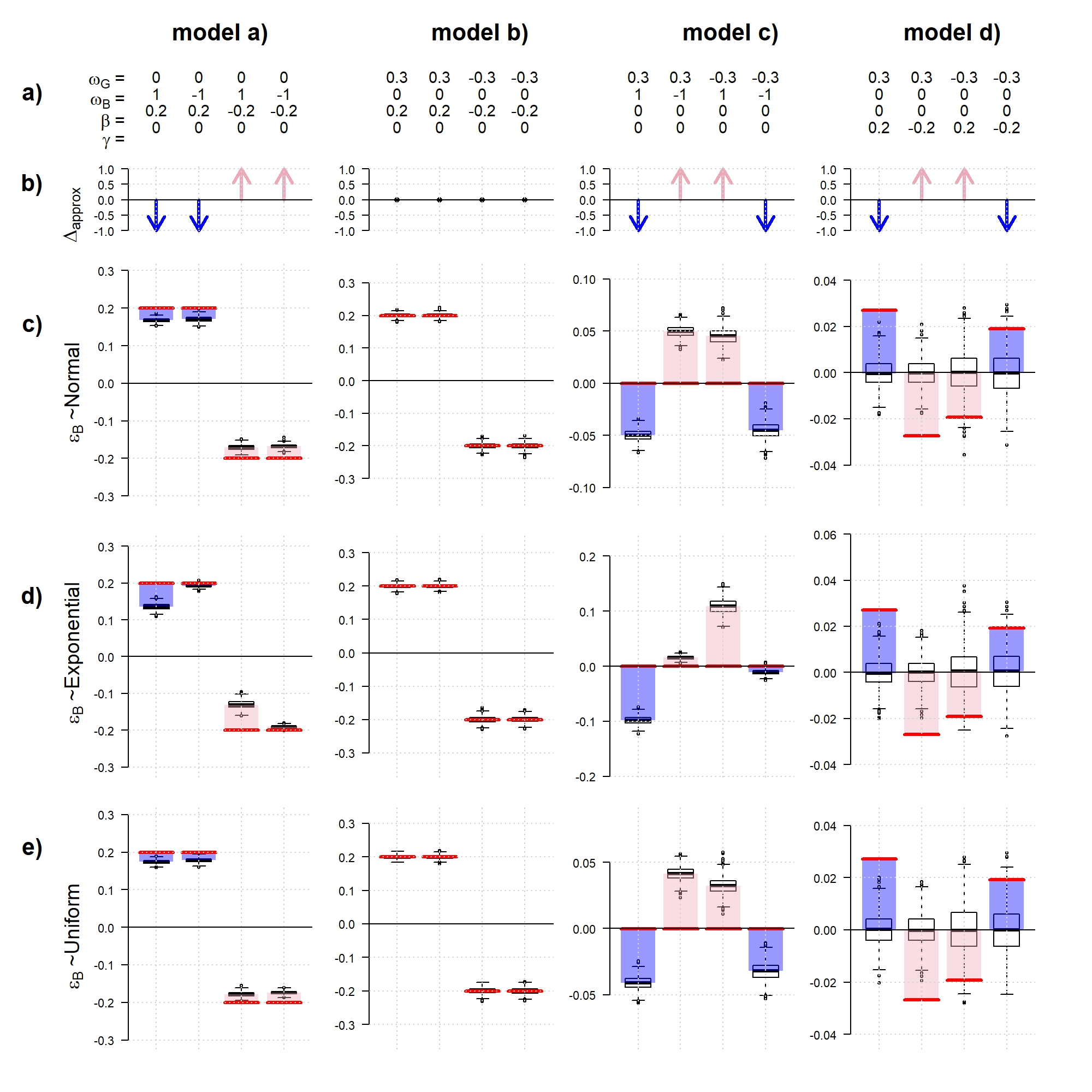

Supplement: S10 Fig — We simulated series of 1,000 replicates each including 100,000 individuals. For each replicate we draw a genetic variant G, a bacterial level B, and a disease status d using the two equations B = βG + γD + εB and E[d|B,G]=1/(1+e−[ω0+ωBB+ωGG]). We use three sets of parameters to match model a (left column), model b (middle column) and models c and d (right column), while changing the direction of the effect. In this simulation, all effects (panel a) were assumed to be large and disease prevalence high (~30%). For each simulation we estimated through standard linear regression the association coefficient between B and G in the whole population and in the cases only. Median of the coefficient in the whole population is indicated by a bold red line, while coefficients observed in cases only are provided in boxplots. The difference between the two coefficients (case only—whole population), Δ, is indicated in blue if negative and in pink if positive. For each set of parameters (panel a), we derived the proposed approximation of Δ (panel b), and the aforementioned estimates while drawing εB from a normal distribution (panel c), an exponential distribution (panel d), and a uniform distribution (panel e). (TIF) [file pgen.1008018.s016.tif]

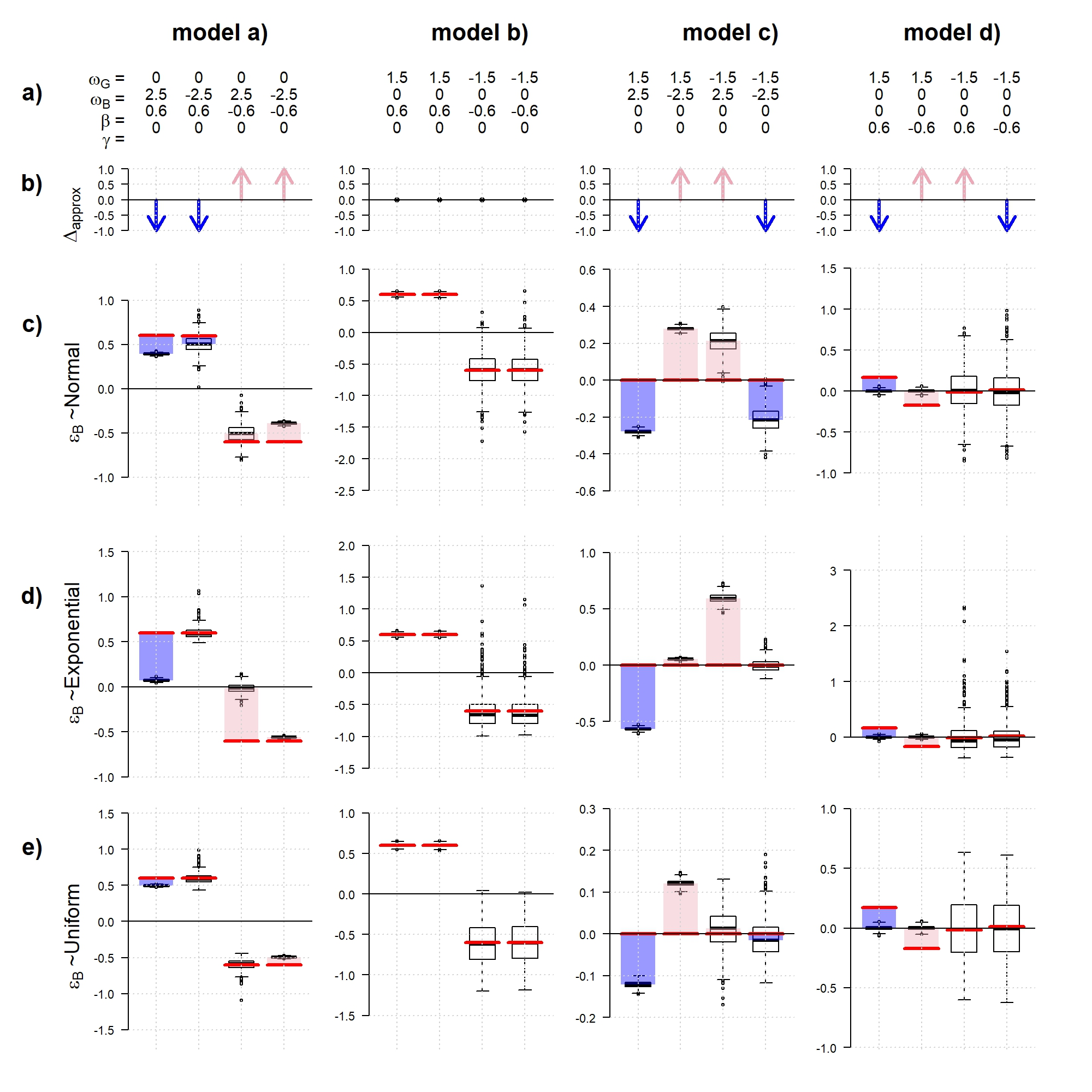

Supplement: S11 Fig — We simulated series of 1,000 replicates each including 100,000 individuals. For each replicate we draw a genetic variant G, a bacterial level B, and a disease status d using the two equations B = βG + γD + εB and E[d|B,G]=1/(1+e−[ω0+ωBB+ωGG]). We use three sets of parameters to match model a (left column), model b (middle column) and models c and d (right column), while changing the direction of the effect. In this simulation, all effects (panel a) were assumed to be large and disease prevalence high (~30%). For each simulation we estimated through standard linear regression the association coefficient between B and G in the whole population and in the cases only. Median of the coefficient in the whole population is indicated by a bold red line, while coefficients observed in cases only are provided in boxplots. The difference between the two coefficients (case only—whole population), Δ, is indicated in blue if negative and in pink if positive. For each set of parameters (panel a), we derived the proposed approximation of Δ (panel b), and the aforementioned estimates while drawing εB from a normal distribution (panel c), an exponential distribution (panel d), and a uniform distribution (panel e). (TIF) [file pgen.1008018.s017.tif]

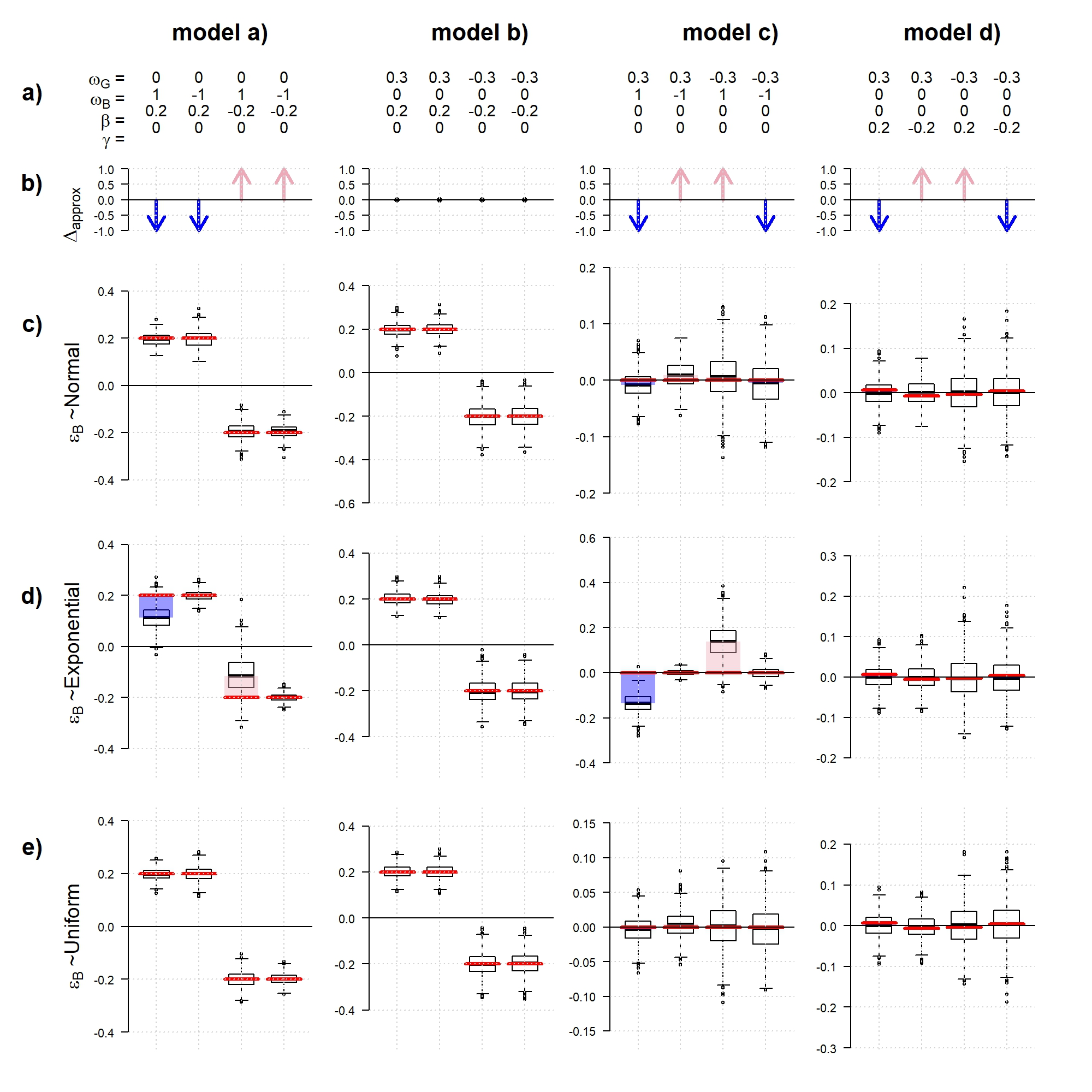

Supplement: S12 Fig — We simulated series of 1,000 replicates each including 100,000 individuals. For each replicate we draw a genetic variant G, a bacterial level B, and a disease status d using the two equations B = βG + γD + εB and E[d|B,G]=1/(1+e−[ω0+ωBB+ωGG]). We use three sets of parameters to match model a (left column), model b (middle column) and models c and d (right column), while changing the direction of the effect. In this simulation, all effects (panel a) were assumed to be large and disease prevalence high (~30%). For each simulation we estimated through standard linear regression the association coefficient between B and G in the whole population and in the cases only. Median of the coefficient in the whole population is indicated by a bold red line, while coefficients observed in cases only are provided in boxplots. The difference between the two coefficients (case only—whole population), Δ, is indicated in blue if negative and in pink if positive. For each set of parameters (panel a), we derived the proposed approximation of Δ (panel b), and the aforementioned estimates while drawing εB from a normal distribution (panel c), an exponential distribution (panel d), and a uniform distribution (panel e). (TIF) [file pgen.1008018.s018.tif]

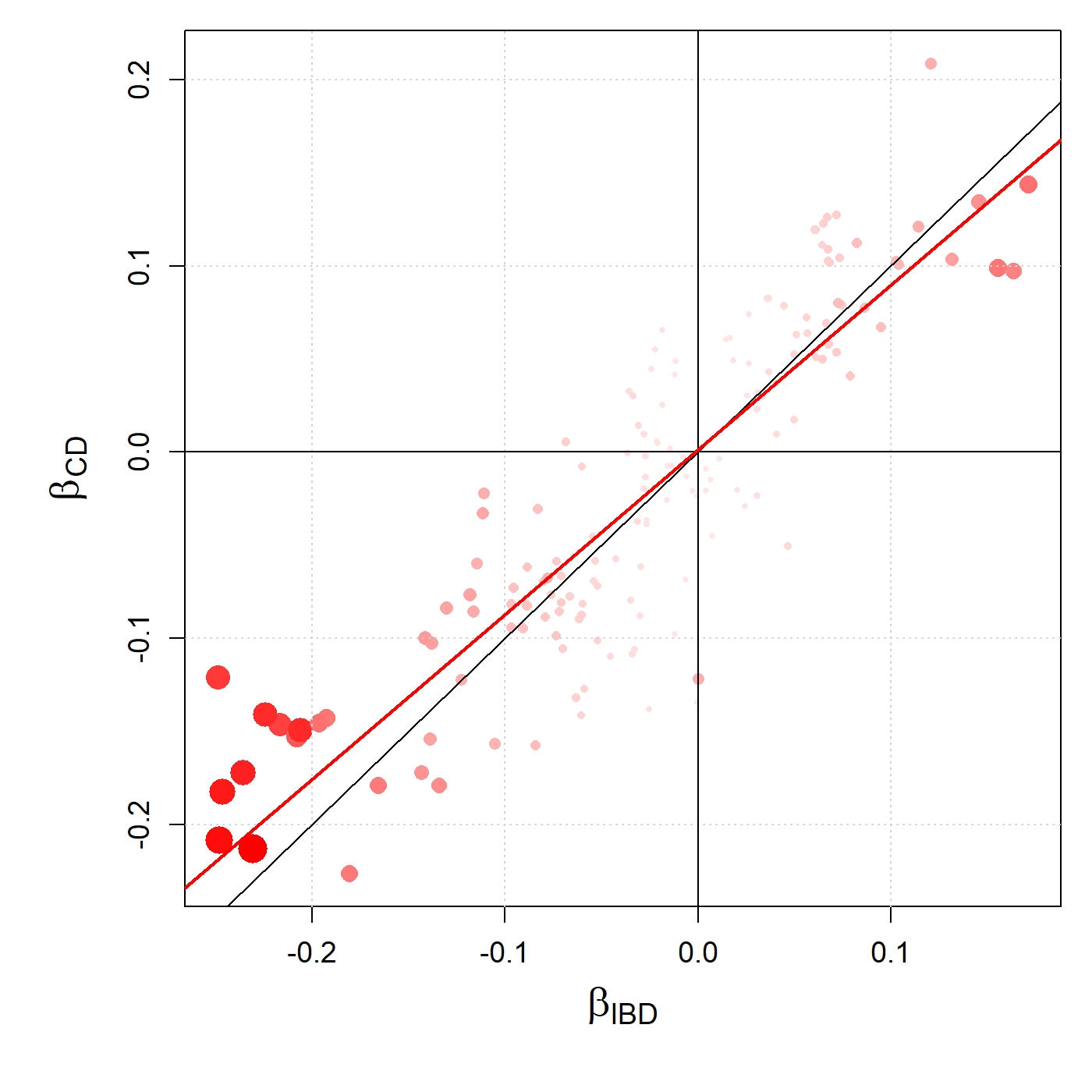

Supplement: S13 Fig — We compared effect estimates for NOD2-bacteria association derived using standard linear regression, after adjusting for confounding factors, in all IBD cases (βIBD) and CD cases only (βCD). The gradient of colors (pink to red) and size of each point (small to large) indicate increasing significance of NOD2-bacteria association in the complete IBD cases dataset. Under the confounding model (b), where CD-bacteria association is partly confounded by a shared genetic effect of NOD2 on both outcomes, the two estimates have the same expectation and therefore the regression slope between the two estimates should be equal to 1 (black line). The observed slope (red line), which equals 0.88, is significantly different from 1 (P = 5e-3, tested using a student t-test assuming an expected slope equals to 1). (TIF) [file pgen.1008018.s019.tif]

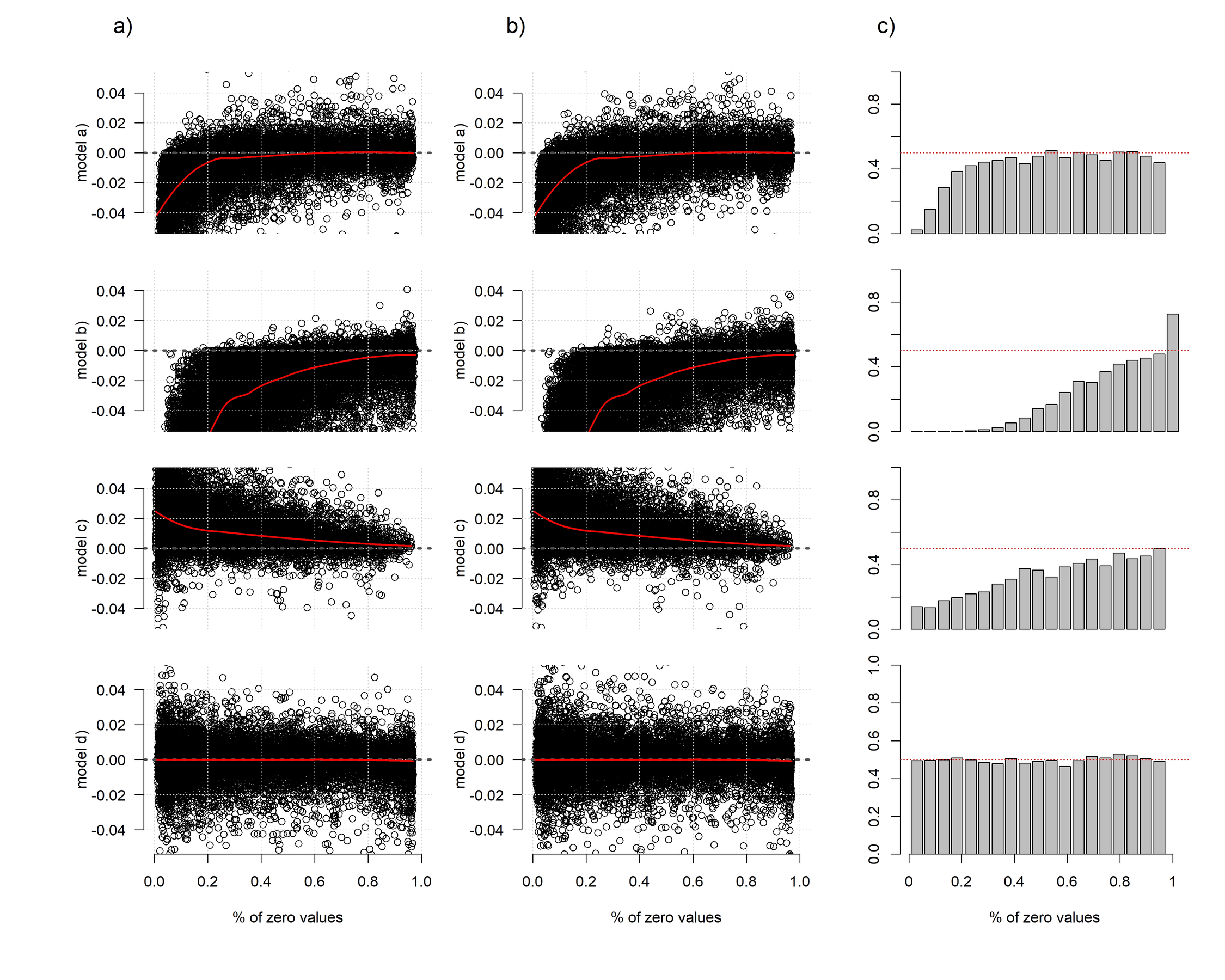

Supplement: S14 Fig — We simulated series of 20,000 replicates each including 10,000 individuals. For each individual, we generated a genetic variant, a bacteria and a case-control status following the four models (a, b, c and d) from Fig 3. The genotype was simulated using a binomial distribution with frequency of the coded allele randomly drawn in [0.05, 0.95]. The bacterial level was simulated using a negative binomial distribution were the dispersion parameters of each replicate were chosen so that the proportion of zero value vary from 0% to 95% across replicates. The case-control status was drawn from a binomial distribution with probability derived using a logit function with parameters matching the each of the four models. For each replicate, a subset of 200 cases was randomly chosen and used to test for association between the genotype and the bacteria using standard linear regression. The left (a) and middle (b) panels show the signed explained variance obtain from this experiment before and after applying an inverse-rank based normal transformation of the bacterial level, respectively. The red line shows the trend derived using local fitting as implemented in R loess function with default parameters. The right panels (c) present median p-values from the latter experiment derived over bins of replicates with a range of percentage of zero-values. (TIF) [file pgen.1008018.s020.tif]

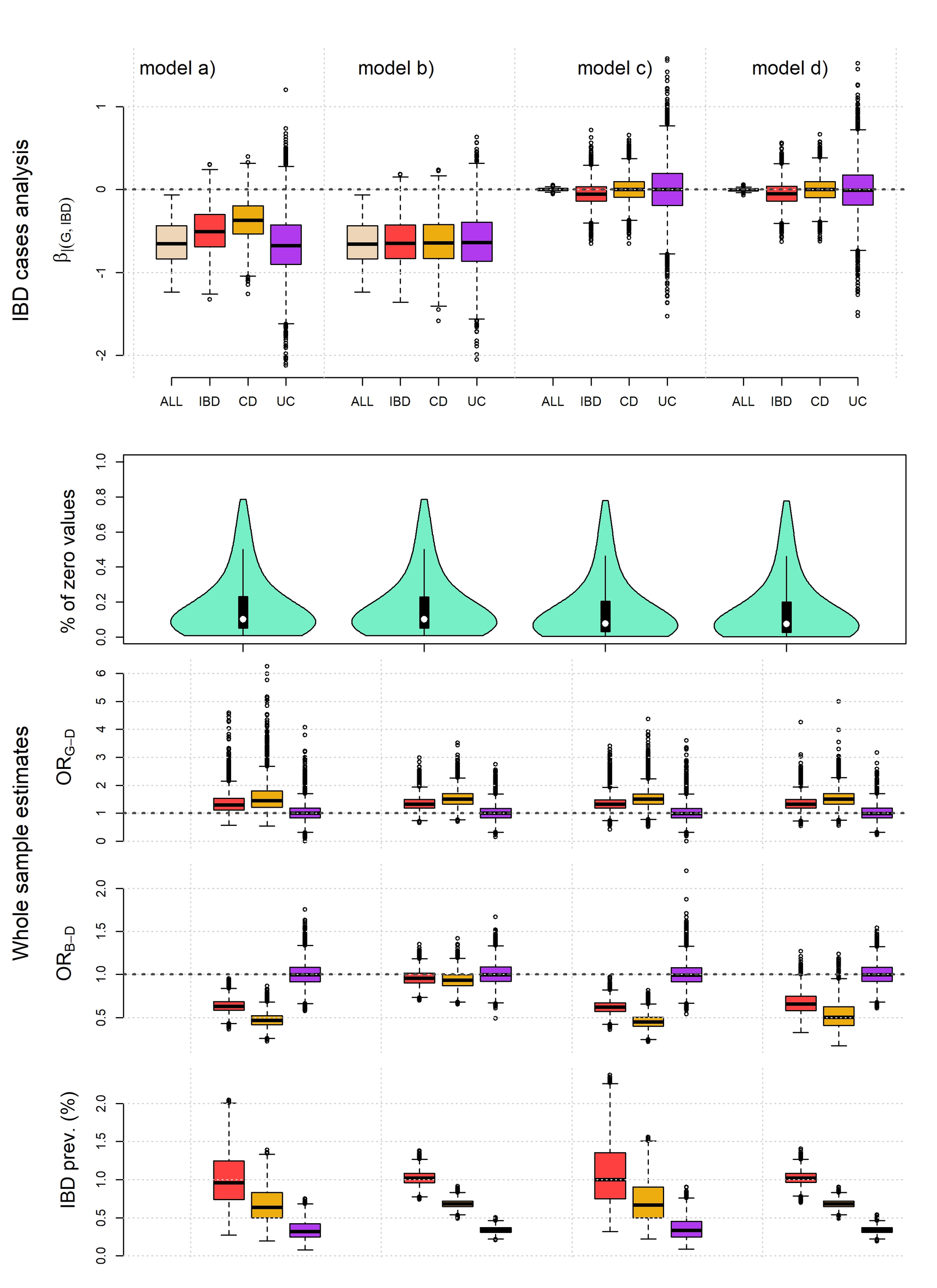

Supplement: S15 Fig — We simulated series of 10,000 replicates each including 30,000 individuals. For each individual, we generated a genetic variant G, a bacteria B and two case-control status, CD, which was defined based on the four causal models (a, b, c and d) from Fig 3, and UC, which was drawn independently of other variables. The two diseases were merged to form the IBD status. The genotype was simulated using a binomial distribution with frequency of the risk allele randomly drawn in [0.05, 0.2]. The bacterial level was simulated using a negative binomial distribution while randomly drawing the dispersion parameter so that the proportion of zero-value ranges in [0%, 80%]. The case-control status was drawn from a binomial distribution with probability derived using a logit function. All parameters from the simulation were set so that it match estimates from the literature and our primary results. The three lower panels show empirical distribution of those parameters estimated in the whole population. Disease prevalence, genetic effect and bacteria-disease association matched both in direction and magnitude for all causal models. We then randomly sampled from each replicate, a subset of 200 cases tested for association between G and B using standard linear regression, and after applying an inverse rank-based normal transformation of B. The test was applied in the sub-sample of IBD cases, but also in the sub-group of CD cases only and the sub-group of UC cases only. (TIF) [file pgen.1008018.s021.tif]

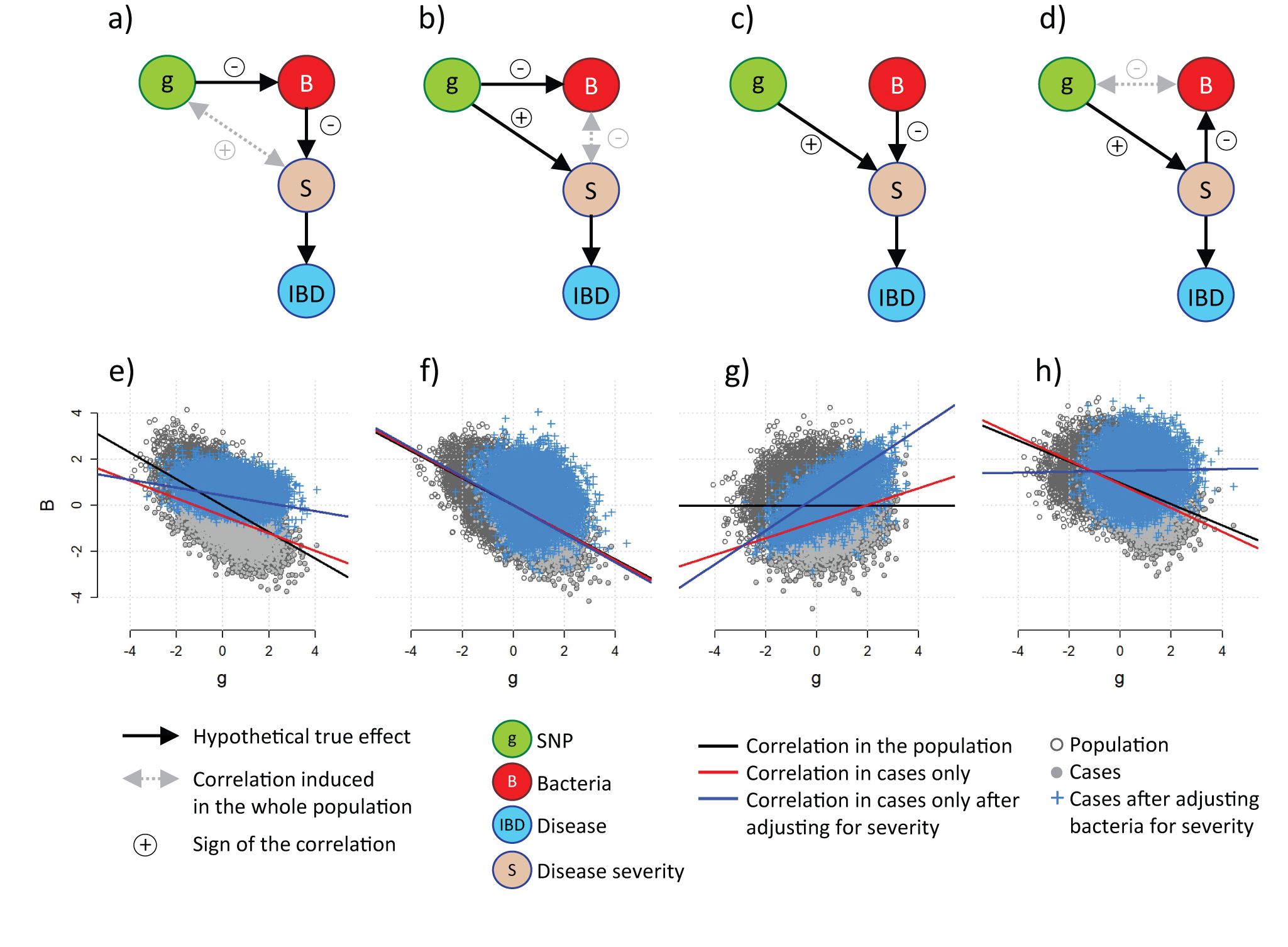

Supplement: S16 Fig — We performed a simulation similar to Fig 3, expect that we replaced IBD status by a severity score. We plotted regression slopes between bacteria and the genetic variant in the whole population, and in cases only, using either the raw bacterial data or after adjusting for severity. Top panels (a, b, c, and d) present the hypothetical causal diagrams and bottom panels (e, f, g, and h) present the corresponding scatterplots of B as a function of g in the population (dark grey points, and trend in black), in cases only (light grey points and trend in red), and again, in cases only but after adjusting the bacteria for severity (blue crosses, and trend in dark blue). In model a) the effect of g on severity is mediated by B; in cases the effect of g on B is underestimated because of the oversampling of participants carrying risk alleles (e). In model b) the genetic variant influences both severity and B, inducing a correlation between severity and B which is observed in both the whole sample and cases only (f). In model c), g and B act independently on severity and are therefore not associated in the population, however g and B are positively correlated in case-only samples because of biased selection (g). Finally, in model d) the effect of g on B is mediated by severity; the indirect association between g and B observed in the general population is still present in cases only, but is canceled when adjusting bacteria for severity (h). (TIF) [file pgen.1008018.s022.tif]

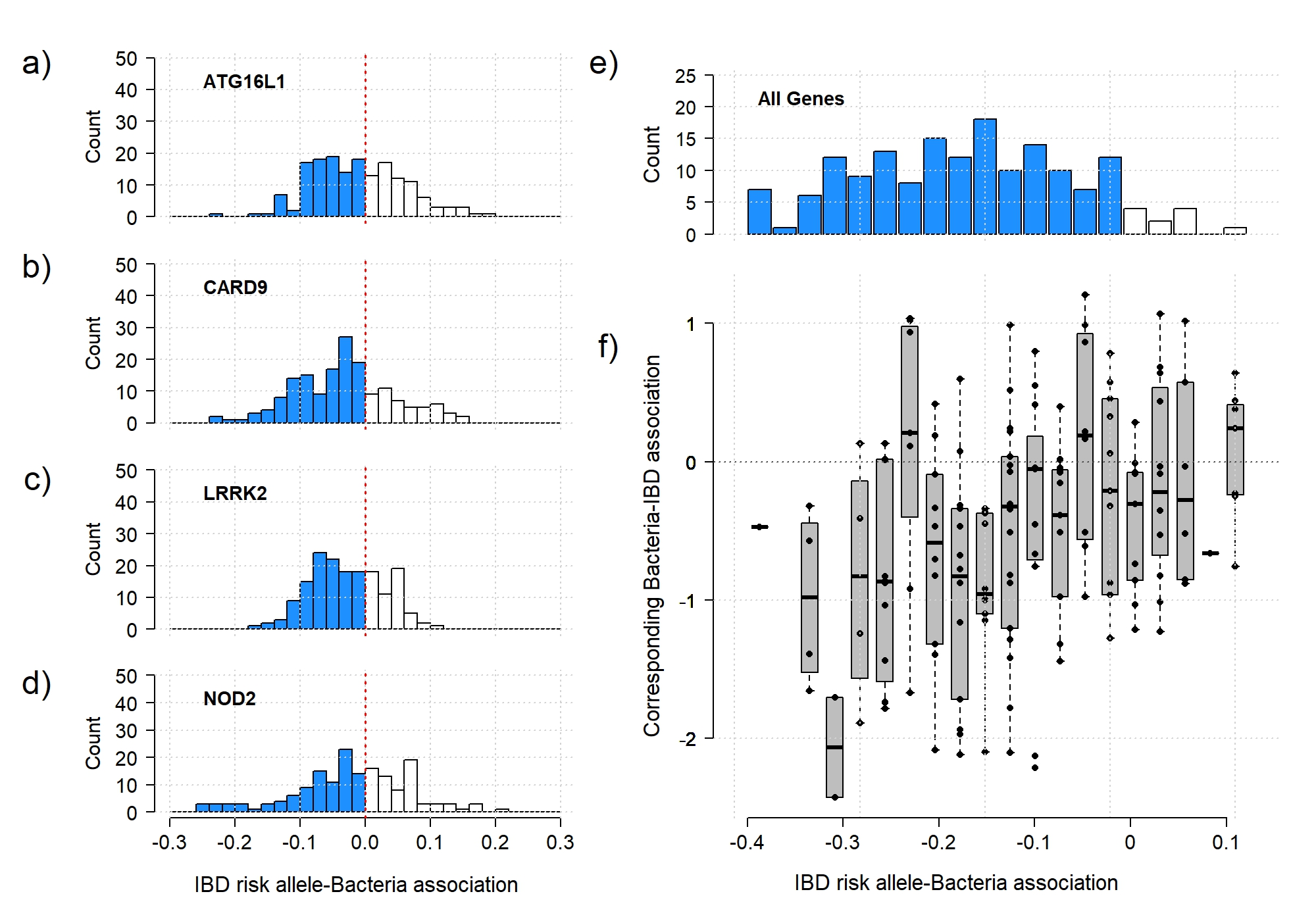

Supplement: S17 Fig — We performed the same analysis as for Fig 4, expect that we adjusted all analyses for flare-remission. The 168 bacterial taxa were tested for association with the variants from each of the four genes considered: (a) ATG16L1 (rs12994997), (b) CARD9 (rs10781499), (c) LRRK2 (rs11564258), and (d) NOD2 (rs2066844, rs2066845, and rs2066847). The histograms on the left panel show the distribution of IBD risk alleles-bacteria association (i.e. of β^g, the regression coefficients) and the enrichment for negative effects (in blue, p-values equal 0.018, 1.3x10^-8^, 9.3x10^-6^, 0.018, respectively). Panel (e) shows a similar histogram while merging the per-risk allele change in bacteria level of the four genes (i.e. summing for each bacteria the β^g of the four genes). Panel (f) shows the distribution of bacteria-IBD association derived in an IBD cases-controls dataset (β^B) for each bin from panel (e). Together, panels (e) and (f) show the strong concordance of the gene-bacteria and bacteria-IBD effects, in agreement with a mediation effect of the risk allele on IBD through the microbiome. In particular, bacteria displaying lower level in carrier of IBD risk alleles are more likely to be negatively associated with the risk of IBD. (TIF) [file pgen.1008018.s023.tif]
